# Spatial distribution of disease-associated variants in three-dimensional structures of protein complexes

**DOI:** 10.1038/oncsis.2017.79

**Published:** 2017-09-25

**Authors:** A Gress, V Ramensky, O V Kalinina

**Affiliations:** 1 Department for Computational Biology and Applied Algorithmics, Max Planck Institute for Informatics, Saarland Informatics Campus, Saarbrücken, Germany; 2 Graduate School of Computer Science, Saarland University, Saarbrücken, Germany; 3 Center for Neurobehavioral Genetics, University of California, Los Angeles, CA, USA; 4 Moscow Institute of Physics and Technology, Moscow Region, Russian Federation

## Abstract

Next-generation sequencing enables simultaneous analysis of hundreds of human genomes associated with a particular phenotype, for example, a disease. These genomes naturally contain a lot of sequence variation that ranges from single-nucleotide variants (SNVs) to large-scale structural rearrangements. In order to establish a functional connection between genotype and disease-associated phenotypes, one needs to distinguish disease drivers from neutral passenger variants. Functional annotation based on experimental assays is feasible only for a limited number of candidate mutations. Thus alternative computational tools are needed. A possible approach to annotating mutations functionally is to consider their spatial location relative to functionally relevant sites in three-dimensional (3D) structures of the harboring proteins. This is impeded by the lack of available protein 3D structures. Complementing experimentally resolved structures with reliable computational models is an attractive alternative. We developed a structure-based approach to characterizing comprehensive sets of non-synonymous single-nucleotide variants (nsSNVs): associated with cancer, non-cancer diseases and putatively functionally neutral. We searched experimentally resolved protein 3D structures for potential homology-modeling templates for proteins harboring corresponding mutations. We found such templates for all proteins with disease-associated nsSNVs, and 51 and 66% of proteins carrying common polymorphisms and annotated benign variants. Many mutations caused by nsSNVs can be found in protein–protein, protein–nucleic acid or protein–ligand complexes. Correction for the number of available templates per protein reveals that protein–protein interaction interfaces are not enriched in either cancer nsSNVs, or nsSNVs associated with non-cancer diseases. Whereas cancer-associated mutations are enriched in DNA-binding proteins, they are rarely located directly in DNA-interacting interfaces. In contrast, mutations associated with non-cancer diseases are in general rare in DNA-binding proteins, but enriched in DNA-interacting interfaces in these proteins. All disease-associated nsSNVs are overrepresented in ligand-binding pockets, and nsSNVs associated with non-cancer diseases are additionally enriched in protein core, where they probably affect overall protein stability.

## Introduction

Human genetic variation ranges from neutral polymorphisms to disease susceptibility variants and pathogenic mutations with high penetrance.^1^ A single individual may carry up to 3 × 10^6^ single-nucleotide variants (SNVs) and up to 3 × 10^5^ insertions and deletions,^2^ but even in disease-affected individuals only few variants of this continuum are expected to be causal, with the rest being neutral. Data on genetic variants that underlie certain disease phenotypes are accumulated in specific databases, for example, ClinVar,^3^ which currently contains >160 000 unique variant records pertaining to 27 261 genes. However, even a strong mutation-phenotype association itself provides no insight into the mechanistic changes to the protein function and/or structure that are caused by the mutation. These changes can result in protein instability or misfolding, or in perturbations of interaction energy, if the affected protein is involved in protein–protein, protein–nucleic acid or protein–ligand interactions.

Computational analysis of the available three-dimensional (3D) structures of human proteins shows that disease-causing missense (non-synonymous) mutations often result in significant alteration of the amino-acid residue properties and disruption of non-covalent bonding.^4^ In contrast, functionally neutral variants tend to be located at the protein surface and to be less conserved than random.^5, 6^ Anecdotal data are available on the involvement of disease-associated missense SNPs in protein–protein interactions (PPI), reviewed in.^7, 8, 9^ A large-scale analysis confirms that disease-related mutations are frequently overrepresented on PPI interfaces.^10^

Several computational methods have been developed to assess the impact of non-synonymous single-nucleotide variants (nsSNVs) on the protein function, with SIFT^11^ and PolyPhen-2^12^ being among the most commonly used ones. Some methods take into account protein sequence-based phylogenetic information pertaining to the mutation,^11, 13^ others rely on the combination of protein structural information, functional parameters and phylogenetic information derived from multiple sequence alignments.^14, 15, 16, 17, 18^ Specific contribution of structural parameters to the prediction performance has been a long-discussed issue.^12, 17^

Numerous tools have been constructed to assess potential changes caused by SNVs in protein 3D structure: SNPeffect database,^18^ for example, ignores the conservation profiles of SNVs and relies on predicted structural features (aggregation, amyloidogenicity, stability) and domain and catalytic site annotations. There are tools that predict the energetic impact of a mutation on the stability of a protein or protein complex.^19, 20, 21, 22, 23, 24^ A thorough comparison and discussion of limitations of these methods can be found in references 17, 25. dSysMap^26^ and Mechismo^27^ extrapolate interactions observed in 3D structures to interactions of homologous proteins. We have recently presented StructMAn, a tool that allows for rapid analysis of large sets of nsSNVs with respect to their impact on interactions of the affected protein with other proteins, nucleic acids and low molecular-weight ligands.^28^

A recent study suggests that considerations related to protein 3D structure can provide hypotheses for molecular mechanisms of action for 40.9% of human mutations that cause inherited diseases.^29^ A large collection of nsSNVs implicated in Mendelian diseases have been experimentally tested for their effect on PPI and two-thirds of them have been found to perturb such interactions.^7^ Using our tool StructMAn, we have recently shown that these mutations are frequently located in potentially functionally important regions of the 3D structures of the corresponding proteins.^28^

In cancer genomics, prediction of the functional impact of genetic variants can be viewed as a search for causative 'driver' mutations among thousands of benign somatic mutations ('passengers') detected in tumor samples and resulting from cancer progression itself.^30^ Both general-purpose and dedicated tools have been applied to study mutations in cancer, for a review see.^31^ For multiple such tools, their ability to predict the effect of cancer-associated mutations has been compared, and the comparison revealed widely varying performance, with methods developed specifically for cancer not outperforming general-purpose tools.^32^ In an analysis of mutations in two somatic cancer samples, one of the general-purpose tools, SNPs3D, has predicted a majority of mutations to have a high functional impact.^13^ The same tool has been recently applied to interpret potential high-risk alleles in complex human disorders in loci identified from GWAS studies.^33^ It was established that 33% of such loci contain at least one nsSNV with a high predicted functional impact. Meta-methods and databases that combine several prediction tools using statistical learning have also been developed.^34, 35, 36^

Cancer3D^37^ maps nsSNVs from The Cancer Genome Atlas (TCGA)^38^ and the Cancer Cell Line Encyclopedia (CCLE)^39^ onto 3D structures of the corresponding proteins and annotates them with respect to predicted cancer driver genes and drug biomarkers. In another study, cancer-associated nsSNVs in common oncogenes and suppressors have been mapped onto 3D protein structures, and the corresponding amino acids have been shown to be enriched in protein interaction interfaces, possibly disrupting them.^40^ Cancer-related mutations have been also shown to form clusters in protein interaction interfaces.^41^

In this study, we use StructMAn for a systematic analysis of amino-acid residues affected by nsSNVs that are associated with different human diseases, as well as common polymorphisms and variants annotated as benign. To do so, we have compiled data sets of cancer-associated nsSNVs and nsSNVs associated with non-cancer diseases from ClinVar,^3^ COSMIC^42^ and UniProt databases,^42^ a set of common nsSNVs from the ExAC (Exome Aggregation Consortium),^43^ and a set of nsSNVs annotated as benign in ClinVar.^3^ Unlike Engin *et al.*,^40^ Cancer3D^37^ and Kamburov *et al.*,^41^ for the cancer-related data set we consider only mutations in 571 genes from Cancer Gene Census, an ongoing effort to catalog those genes, for which mutations have been causally implicated in cancer,^42^ to ensure high relevance of these mutations to oncogenesis. We also extend analysis of Cancer 3D^37^ and Engin *et al.*^40^ by considering interactions with low molecular-weight ligands and DNA, and that of Kamburov *et al.*^41^ by taking into account structures of homologous proteins.

The structural coverage of the human proteome is still low: 28% of distinct genes corresponding to proteins in the UniProt human reference proteome, as of September 2016, have an available 3D structure resolved for at least a part of the protein sequence, but only 8.4% have a structure that covers over 90% of the sequence length. Considering structures of proteins that are homologous to human proteins allows to put many more nsSNVs into structural context. As the interactions in homologous proteins are conserved down to relatively low sequence identity,^44^ the structural context of the corresponding residues in such structures, also called templates, is likely to be the same for human proteins affected by nsSNVs. The selection of templates in StructMAn is performed in such a way that they can later be used for homology modeling of the corresponding proteins and mutations in them. It must be also noted that we do not assess the impact of nsSNVs on the protein overall stability, but rather focus on those that modulate specific protein interactions.

## Results

### Overview of the data sets

By merging variation data from various sources (see Materials and methods for details) we were able to compile comprehensive SNV data sets (Table 1). To the best of our knowledge, here for the first time, we consider cancer-associated germline, and cancer-associated somatic mutations separately, as well as mutations associated with non-cancer diseases along with common polymorphisms and benign ClinVar variants. Germline cancer mutations segregate in families and confer predisposition to inherited cancer syndromes.^45^ They are associated with only 5–10% of all cancers and typically act in dominant mode with high penetrance. Somatic mutations are present only in the cancer tissues and, compared with the germline ones, are much more numerous and diverse (Table 1). The data set for mutations associated with non-cancer diseases is almost four times larger than all cancer-associated mutations taken together, and only half of the size of the set of common variants. Cancer-associated nsSNVs, in turn, are comparable in number to benign ClinVar variants. With the sizes of data sets reaching tens of thousands, statistical analysis of structural features of amino acids corresponding nsSNVs becomes amenable. We believe these data to comprise the largest and the most diverse data set of nsSNVs ever subjected to structural analysis. For each of these data sets we have created a randomized control data set, in which the identity of the genes and the number of nsSNVs per gene were kept the same, but the nsSNVs were randomly introduced into the nucleotide sequence.

Annotation of structural context for these disease-associated mutations opens new possibilities for analysis of their functional impact. While experimentally resolved 3D structures are available only for ~19–73% of proteins, depending on the data set (Table 1), our modeling procedure allows for reconstructing 3D structures for 51–100%. More proteins from the disease-related data sets can be characterized by structural models than in the set of common variants, probably due to greater interest in these genes in the scientific community. Of the whole human proteome, 32.7% of all proteins and 18.5% of all residues could be mapped to a structural template using the same parameters.

**Figure 1:**
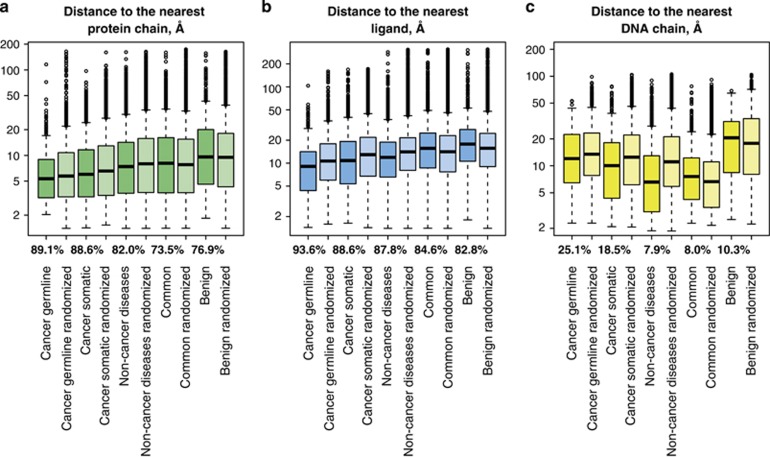
Distance between residues corresponding to nsSNVs and the nearest interaction partner (log scale). Biological data sets are shown in a darker shade. The fraction of mapped nsSNVs, for which a template with a co-resolved corresponding interaction partner is provided below boxes representing distribution of distances to protein, ligand and DNA interaction partners for each biological data set. For randomized data sets, all 10 replicas are used to create the plots. (**a**) Distances to the nearest protein chain. (**b**) Distances to the nearest ligand. (**c**) Distances to the nearest DNA chain.

Moreover, a large fraction of nsSNVs could be mapped into protein–protein, protein–ligand or protein–DNA complexes (Figure 1). The fraction of nsSNVs, for which at least one template with a specific interaction partner can be found is in the range 70–90% and rather stable across all data sets, except for DNA-containing complexes, which are relatively scarce, and only correspond to ~20–25% of all nsSNVs in cancer-associated sets. Somewhat fewer variants can be mapped into templates containing interaction partners for randomized data sets: the drop is two- to fivefold, between 50 and 80% of variants for protein and ligand interactions, and between 4.6 and 9.5% for DNA interactions. Together with Table 1, this indicates that in the randomized data sets quite many variants do not map to any protein with a resolved 3D structure, and even fewer map to complexes with other molecules, which is in agreement with the discussion of nsSNVs in disordered regions below.

**Figure 2:**
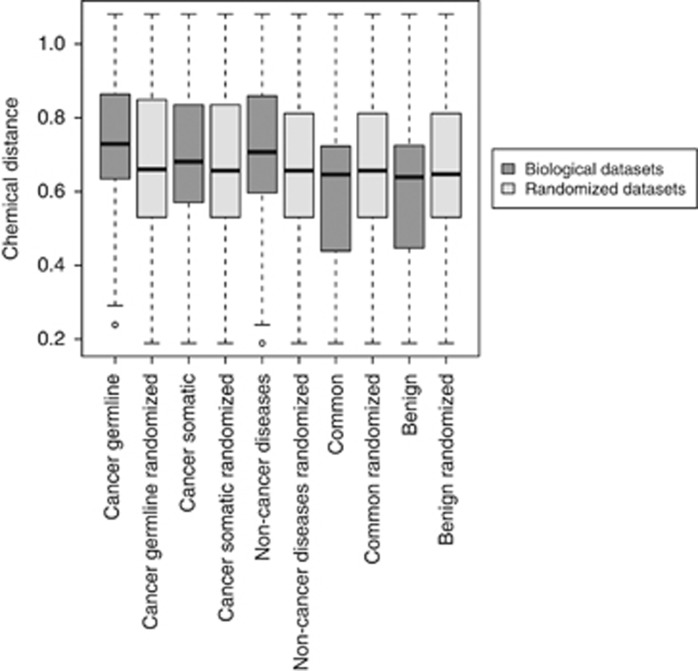
Chemical difference between wild-type and mutated residues. Gray bars indicate biological data sets, light-gray bars indicate randomized data sets. Chemical distance is calculated as Euclidean distances between the end points of the vectors representing five most important numerical descriptors of physical and chemical properties^80^ of the wild-type and mutant amino acids.

Studied sets of mutations also differ in the chemical properties of the substitutions calculated as the average chemical distance and average BLOSUM62 similarity score between each wild-type amino-acid residue and all possible mutations (Figure 2, Supplementary Figure S1). All sets of disease-associated mutations have a higher degree of chemical dissimilarity and median BLOSUM62 scores below zero, indicating events that are likely to change the chemical properties of the involved protein sites substantially. Hereafter by 'disease-associated' nsSNVs we mean cancer-associated nsSNVs as well as nsSNVs associated with non-cancer diseases collectively, unless a particular set is explicitly specified.

We have estimated the fraction of nsSNVs corresponding to residues in disordered regions with IUPred.^46^ Such predictions are based on protein sequence and thus available for variants not necessarily mapped to 3D structure. The predicted disordered fraction is higher among common and benign nsSNVs (30.3% and 28.2%, respectively) compared with disease-associated nsSNVs (10.4, 9.3 and 5.4% for germline cancer-associated nsSNVs, somatic cancer-associated nsSNVs, and nsSNVs associated with non-cancer diseases, respectively, Supplementary Table S1). In the randomized sets, between 14 and 30% of all nsSNVs are predicted to be disordered. For nsSNVs that can be mapped onto protein 3D structures, these values are closer: between 5.5 and 9.3% for all data sets, which indicates that most nsSNVs in disordered regions from the sets of common and benign variants cannot be mapped onto experimentally resolved 3D structures, in agreement with the natural bias of experimentally resolved structures towards compact domains. In disease-associated sets, fewer positions are predicted to be disordered than in the corresponding randomized data sets, whereas for common and benign variants it is the other way around or close to equal values. This trend is also observed for nsSNVs mapped into resolved 3D structures.

When analyzing the distribution of the corresponding positions in the template structures with respect to the elements of secondary structure with DSSP^47^ using a majority vote over all available template structures, we find no significant trends related to pathogenicity of the corresponding sets: overall, between 32.5 and 43.1% of positions corresponding to nsSNVs lie in turns, bends, coils or isolated beta-bridges, whereas between 34.7% and 43.1% in helical and between 21.3 and 26.2% in extended sheet structures (Supplementary Table S1).

### Spatial distribution of nsSNVs

**Figure 3:**
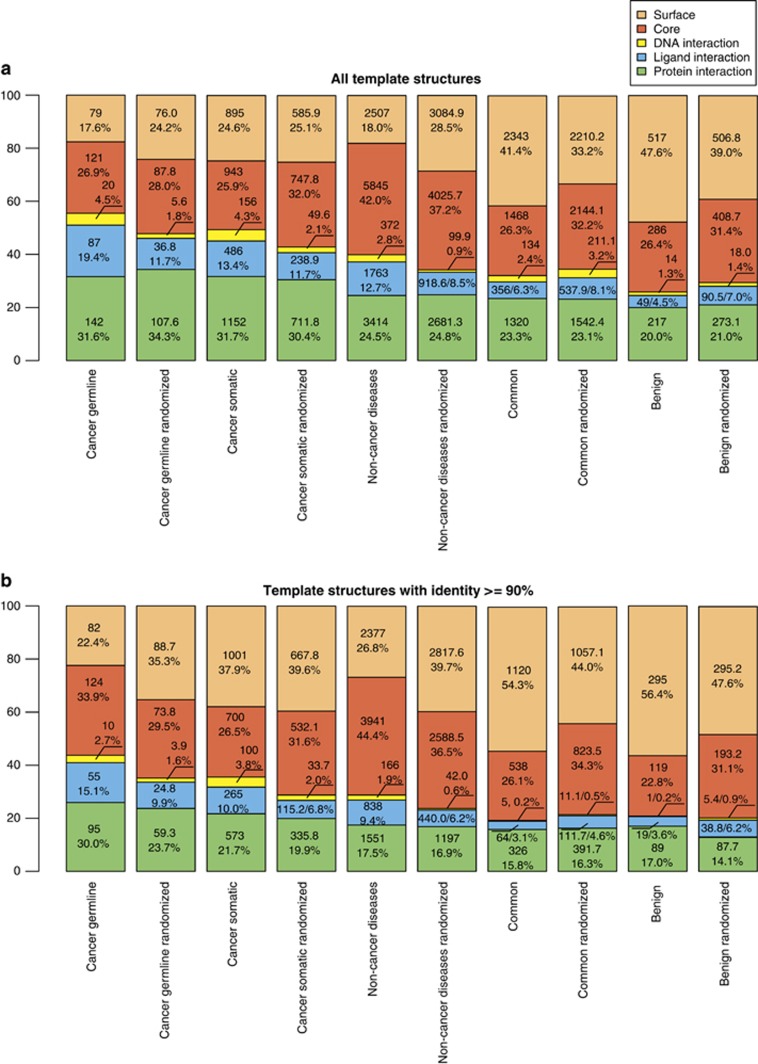
Spatial distribution of nsSNVs in the analyzed data sets. For randomized data sets, mean values over 10 replicas are used. (**a**) For templates with ⩾35% sequence identity. (**b**) For templates with ⩾90% sequence identity.

We have annotated our data sets with respect to location of the corresponding amino acid in the protein 3D structure: each nsSNV was assigned to be either at the protein surface not contacting another molecule, or buried in the protein core, or on a protein–DNA, protein–protein or protein–ligand interaction interface defined as being closer than 5 Å to the respective interaction partner. In case when a residues lies closer than 5 Å to more than one interaction partner of different kinds, the corresponding nsSNV was assigned the class where the distance was lowest (Figure 3). We found very few protein–RNA contacts, and did not analyze this class of interactions further. When all contacts of a residue were taken into account, the trends of the overall spatial distribution among different data sets remain the same as for mutually exclusive classes (Supplementary Figure S2). To ensure statistical significance of the observations, we performed bootstrapping by sampling with replacement each data set 1000 times. In all cases standard deviation over 1000 samples is smaller than difference between fractions of nsSNVs corresponding to the same contact class across different data sets (Supplementary Table S2).

The total number of templates with sequence identity to the mutation-carrying protein >90% is much lower than for the identity threshold of 35%, namely 31 012 vs 348 730. However, the relation between the fractions of nsSNVs falling into different classes of spatial distribution is not significantly different from that for the complete sets (Figure 3), in line with previously reported conservation of PPI interfaces.^44^ This also ensures stability of our results with respect to the alignment quality: highly similar templates produce high-quality alignments, and the distribution of structural classes for such templates is qualitatively identical to templates with alignments of varying quality. Thus aggregated conclusions of analysis of spatial distribution of the corresponding amino acids do not depend on the evolutionary distance to any homologous template. Hence further we will use only the general set with identity threshold of 35% in our analysis.

All disease-associated sets of nsSNVs have a high fraction of mutations corresponding to amino-acid residues that contact other molecules in protein complexes compared with the set of common and benign variants (55.5, 49.4 and 40.0% for cancer-associated nsSNVs germline and somatic and nsSNVs associated with non-cancer diseases vs 32.0 and 25.8% for common and benign variants).

Compared with all other sets, the fractions of protein–protein contacts are high in cancer-associated data sets. This is apparently in line with previously observed trend of cancer-associated mutations to be overrepresented in PPI interfaces.^40^ However, exactly the same trend is observed for the corresponding randomized sets, which casts doubt on the proposition that this is a special property of cancer-associated nsSNVs. An alternative explanation could be that genes carrying cancer-associated nsSNVs have more PPI interfaces, which is in line with the observation that such genes frequently act as hubs in PPI networks.^48^ However, it has been previously reported that correction for number of experimental measurements available for a protein may render this effect insignificant.^49^ As structures of cancer and other disease-associated proteins have been studied very intensively (Table 1), we corrected for this bias by sampling from the sets of common and benign nsSNVs those with the same numbers of identified protein templates as in disease-associated data sets. For such sampled sets, the proportion of nsSNVs mapped to PPI interfaces is not significantly different from that in the 'true' disease-associated data sets (Supplementary Table S3). This suggests that neither disease-associated nsSNVs are enriched in PPI interfaces, nor disease-associated proteins have more such interfaces than proteins harboring neutral variants.

The median distances from structurally mapped nsSNVs to the closest protein chain lie between 5 and 10 Å for all data sets, meaning that over a half of such nsSNVs do not directly contact them. However, the distributions of these distances are significantly shifted towards lower values for all disease-associated classes compared with both common and benign variants (*P*-value in two-sided Wilcoxon test is 2.053e-14 for germline cancer-associated mutations, <2.2e-16 for somatic cancer-associated mutations, and 0.002999 for mutations associated with non-cancer diseases compared with common variants, respectively; for all disease-associated sets, the *P*-value of these distances compared with benign variants is <2.2e-16, Supplementary Table S4).

Disease-associated nsSNVs are enriched in ligand contacts, in contrast to the corresponding randomized sets, which indicates that position within the harboring protein is crucial for these nsSNVs, apparently unlike the case of protein–protein contacts. This effect is significant even after correction for the number of available templates (Supplementary Table S3). Distributions of distances to the nearest ligand are significantly shifted toward lower values in all sets of disease-associated mutations compared with the common and random sets (*P*-values are < 2.2e-16 for all these data sets compared with common and benign variants). It must be noted that for this analysis we do not distinguish between natural ligands and drug-like molecules. A more detailed analysis of oncogenes and tumor-suppressor genes (TSG) (see below) shows that removal of these molecules does not qualitatively affect the results. The particular trend to lower distances can be explained both by tendency of disease-associated mutations to disrupt protein function by altering a specific ligand-binding site, as well as by drug-like molecules being designed to target sites where such mutations occur (see further discussion of drug-like molecules in contact with oncogenes and tumor-suppressor genes below).

Cancer-associated sets also have a higher fraction of mutations corresponding to DNA-contacting residues, in agreement with the fact that DNA repair pathways are often distorted in cancer^30^ (see also pathway enrichment analysis below). Compared with the corresponding randomized data sets, all disease-associated variants are enriched in nsSNVs in the DNA contact class, particularly cancer-associated ones (4.5% vs 1.8% and 4.2% vs 2.1%, respectively). The common and benign variants are slightly depleted of such nsSNVs. Whereas only 7.9% of nsSNVs associated with non-cancer diseases map into 3D complexes with DNA (as opposed to 25.1% in germline cancer-associated and 18.5% in somatic cancer-associated mutation sets), the distribution of distances to DNA is markedly shifted to lower values compared with both cancer-associated data sets (*P*-values 5.721e-07 and <2.2e-16, respectively) with a median distance of 6.58 Å (compared with 12.03 Å for germline and 10.02 Å for somatic mutations). These mutations are likely to alter specific interactions with DNA. nsSNVs that can be mapped in DNA-containing complexes from cancer-associated data sets tend to rather lie in protein core, thus probably destabilizing it as can be exemplified by mutations in the p53 core,^50, 51^ or on PPI interfaces.

Interestingly, the fraction of mutations that correspond to the residues in the protein core is lower in the sets of cancer-associated nsSNVs than in the set of nsSNVs associated with non-cancer diseases (26.9% for germline and 25.9% for somatic mutations vs 42.0%), almost at the same level as among common and benign variants (25.9% and 26.4%, respectively). In the corresponding randomized sets this trend is supported: common and benign variants are depleted of core nsSNVs compared with their randomized version, and variants associated with non-cancer diseases are enriched in such positions.

Very few mutations of disease-associated classes correspond to residues on protein surface that do not take part in any contact (17.6%, 24.6% and 18.0% as opposed to 40.8% of common and 47.7% of benign nsSNVs). Common and benign variants are, on the other hand, enriched at the protein surface compared with all disease-associated nsSNVs, but are depleted in interaction interfaces with all kinds of investigated molecular partners, which is supported by the corresponding randomized sets. Thus they represent a relatively harmless type of mutation from the structural point of view: a surface residue that is not in any kind of functionally relevant contact can be mutated without much consequence.

### Protein complexes with multiple mutated subunits

**Figure 4:**
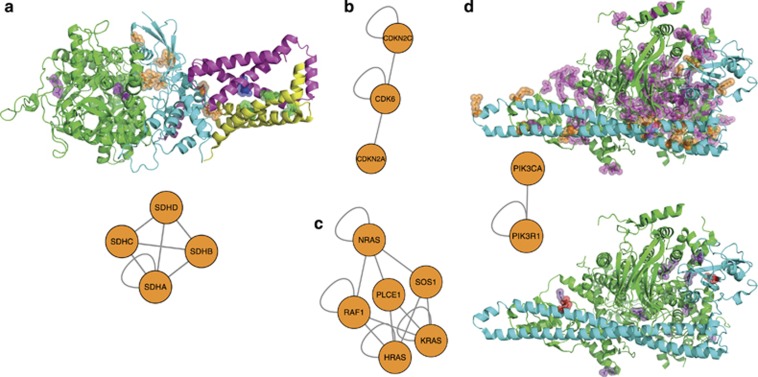
Protein complexes with nsSNVs in multiple subunits. (**a**) Mitochondrial respiratory complex II (mapped onto a homologous complex from porcine heart, PDB id 1ZOY) and the corresponding sub-network (see text). FAD-binding protein is shown in green, mutations therein in pink; iron–sulfur protein is shown in cyan, mutations therein in orange; large cytochrome binding protein is shown in magenta, mutations therein in purple; small cytochrome binding protein is shown in yellow, mutation therein in limegreen. In the sub-network, nodes correspond to individual proteins, edges depict interactions between them. (**b**) Sub-network corresponding to complexes of CDK6 with its inhibitors CDKN2A and CDKN2C. Stoichiometry of the complexes is not accounted for, and nodes with a single loop edge correspond to associations of multiple identical subunits. (**c**) Sub-network corresponding to NRas, KRas and HRas and their downstream kinase RAF1 and activity factors SOS1 and PLCE1. (**d**) PIK3CA-PIK3R1 complex with mutations corresponding to cancer-associated somatic nsSNVs (top) and to nsSNVs associated with non-cancer diseases (bottom), PDB id 4L1B and the PIK3CA-PIK3R1 sub-network. PIK3CA subunit is shown in green, mutations therein in magenta and purple. PIK3R1 subunit is shown in cyan, mutations therein in orange and red.

There are several cases in our data when disease-associated mutations can be found in multiple subunits of the same protein complex, thus forming networks of mutated interacting proteins (Figure 4, Supplementary Figures S3–S5). The whole network of such complexes shows a clear preference to homooligomers (that is, complexes of several identical protein chains) (Supplementary Figures S3–S5), but includes some notable examples of more complex assemblies. For example, in the mitochondrial respiratory complex II, we find cancer-associated germline mutations in all four subunits (Figure 4a). These mutations are located significantly more closely to each other than all other pairs of residues of these proteins (*P*-value=0.001567). Another example is a sub-network corresponding to interactions of CDK6 with its inhibitors CDKN2A and CDKN2C (Figure 4b), or between membrane-associated GTPases NRas, KRas and HRas and their downstream kinase RAF1 or activity factors SOS1 and PLCE1 (Figure 4c). In the heterodimer of PIK3CA and PIK3R1 (Figure 4d), in which both subunits carry cancer-associated somatic mutations, which lie also significantly more closely to each other than other pairs of residues of the two subunits (*P*-value <2.2e-16). The same complex, as well as PIK3CA-PIK3R2, PIK3CD-PIK3R1 and PIK3CD-PIK3R2, carries mutations associated with non-cancer diseases, which are also significantly closer to each other than on average (*P*-value=0.04133). However, in a comparison of distances between the mutated residues and all pairs of residues in all homo- and heterooligomers, we do not observe a significant trend of disease-associated mutations to be closer to each other than on average.

### Contacts of mutated residues in oncogenes and TSG

Certain cancer-associated genes are commonly called oncogenes or TSG, reflecting their role in disease progression.^30^ Using classification from the COSMIC Cancer Census Genes, we identified eight oncogenes and nine TSGs among proteins with germline mutations, and 53 oncogenes and 30 TSGs among proteins with somatic mutations. Seventy-two proteins with germline mutations and 159 proteins with somatic mutations cannot be attributed to either class.

**Figure 5:**
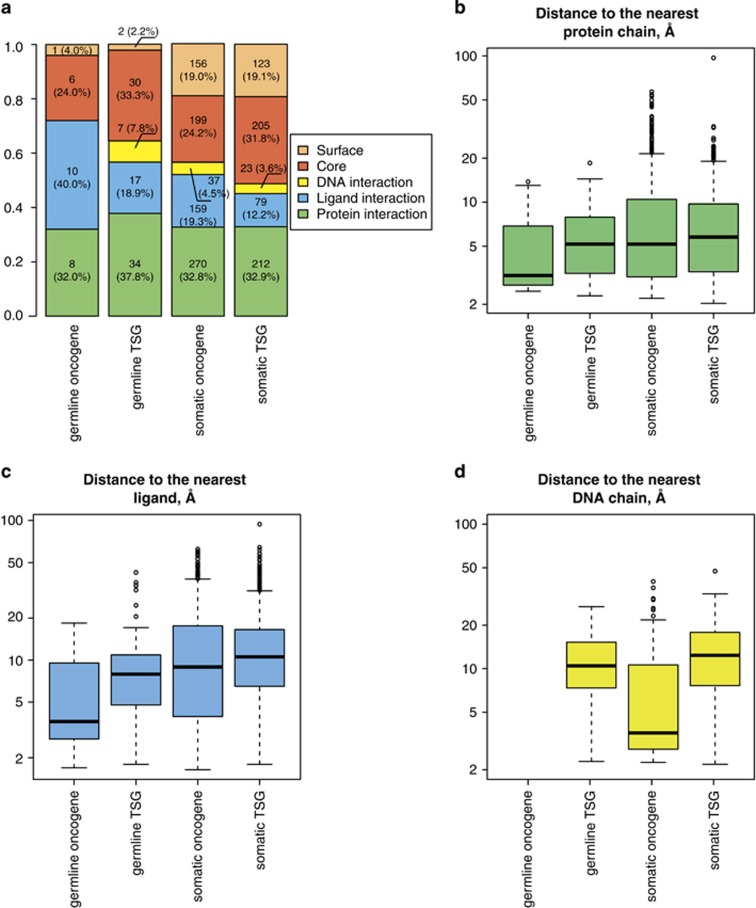
Contacts and distance distributions for oncogenes and tumor-suppressor genes (TSG). (**a**) Distribution of nsSNVs into structural classes. (**b**–**d**) Distances to the nearest interaction partners: (**b**) protein chain, (**c**) ligand, (**d**) DNA chain.

The number of mutations required to unleash tumor progression has been reported to differ between the two groups of genes,^30^ so we investigated mutations in them separately (Figure 5, Supplementary Table S5). Of germline mutations, we could map only 25 from oncogenes and 90 from TSGs into 3D protein structures, and the corresponding values of standard deviation in the bootstrap analysis are quite large, but for somatic mutations, we could map 823 and 646 from oncogenes and TSGs, respectively, and the values of standard deviation lie within 1–2% in all contact classes (Supplementary Table S5). We observe that the fraction of mutations located in the protein core is higher for TSGs than for oncogenes among the somatic mutations (24.5% vs 11.8%) and also exceeds that in the overall sets of germline and somatic cancer-associated mutations (20.7% and 16.2%, respectively). The observation is in agreement with the expectation that somatic mutations in TSGs may implement their pathogenic effect by 'knocking out' harboring proteins.^52^

For oncogenes, we observe an enrichment of protein and small-molecule contacts, both among germline and somatic mutations. Sixteen of 23 (69.6%) germline and 373 out of 760 (49.1%) of somatic nsSNVs correspond to mutations that lie within 5 Å from another protein chain, which means that they can directly influence contact specificity and binding affinity. However, this enrichment cannot be confirmed when the correction for the number of available structural templates is taken into account (cf. The discussion in 'Spatial distribution of nsSNVs' above): the number of nsSNVs from neutral data sets residing in protein with the same distribution of the number of templates is frequently even larger for both germline and somatic mutations in oncogenes and TSGs (Supplementary Table S5).

Fourteen of 25 (56.0%) of germline and 242 of 787 (30.7%) of somatic mutations in oncogenes lie within 5 Å from a small-molecule ligand (a single mutation can lie within 5 Å from both a ligand and another protein chain, which introduces ambiguity into the functional interpretation of these findings). The median distance to the nearest ligand is higher in the general set of somatic cancer-associated mutations (10.8 Å) than for somatic mutations in oncogenes (8.9 Å), and there is a significant difference between these distributions (*P*-value=6.622e-06).

Low molecular-weight ligands can be found in template complexes for almost all cancer-associated mutations in oncogenes and TSGs (Figure 5c). Some of these ligands are specific anticancer drugs or drug-like molecules. To investigate the location of mutations in naturally occurring complexes, we have excluded all ligands listed as drugs in DrugBank^53^ from consideration, and the fractions of nsSNVs classified as ligand contacts drop by one, two, and three nsSNVs for germline nsSNVs in oncogenes, somatic nsSNVs in oncogenes and TSGs, respectively. In the first two cases nsSNVs are reclassified as protein contacts, and, in case of somatic nsSNVs in TSGs, all three nsSNVs in SETD2 are reclassified as core mutations (these residues are in contact with S-adenosylmethionine, which is listed in DrugBank, in the structure of a homologous histone-lysine *N*-methyltransferase NSD1, Protein Data Bank (PDB) id 3OOI). This may explain pathogenicity of these nsSNVs in patients not previously exposed to treatment. Additionally, we excluded all ligands bound to tyrosine kinases ABL1, ALK, BTK, CSF1R, DDR1, EGFR and KIT, as they represent important oncogenes targeted by new-generation inhibitors.^54^ This leads to a milder effect: one somatic nsSNVs Ala366Val in ABL1 is reclassified from ligand-contacting to core.

Interestingly, somatic mutations in oncogenes are shifted to DNA-binding interfaces (median distance from DNA 3.59 Å putting 56.6% of all such mutations below the 5 Å contact threshold), which is not the case either for somatic mutations in TSGs (median distance to DNA 12.36 Å, distance distributions differ with a *P*-value of 3.327e-09), nor for the general set of somatic cancer-associated mutations (median distance 10.05 Å, *P*-value 4.461e-06) (Figure 5d). Our correction for the number of available templates also confirms that this enrichment is significant (Supplementary Table S5). The median distance to DNA in all control data sets that were sampled either from common or benign nsSNVs and preserve the distribution of the number of templates for the harboring proteins as for somatic mutations in oncogenes is between 9.79 Å and 10.89 Å. Most of the amino acids corresponding to somatic nsSNVs in oncogenes reside in domains of typical DNA-binding folds, such as zinc fingers, basic leucine zippers or homeodomains. Observed wild-type residues are usually characteristic for these folds (for example, cysteines in zinc fingers) or typical DNA-interacting residues such as arginine. It is plausible that mutations of these residues are essential for maintaining DNA contacts in all proteins with a particular fold, or in some cases, such as Zn-binding cysteines in Zn fingers, for maintaining a fold *per se*.

Fraction of nsSNVs in predicted disordered regions are consistently lower in oncogenes and higher in TSGs, both for germline and somatic mutations (4.8 and 4.0% in oncogenes, 16.6 and 11.5% for TSGs vs 10.4 and 9.3% for the general germline and somatics sets, respectively). In addition, TSGs are enriched in mutations in protein core, and both oncogenes and TSGs are depleted of mutations on protein surface.

### Pathway and GO-term enrichment analysis

For each set of disease-associated mutations, we performed differential analysis of pathways from the Reactome Pathway Database^55^ associated with the corresponding proteins (Table 2). In this analysis we selected pathways that are overrepresented in the set of genes affected by disease-associated nsSNVs compared with genes with common variants. We do this by summing the candidate scores for all nsSNVs in all genes from a particular pathway and comparing this value to the corresponding value calculated for common variants. This analysis summarized cellular processes particularly affected by mutations from a certain category. Not surprisingly, germline cancer-associated mutations are enriched in proteins involved in DNA repair and stress response, somatic cancer-associated mutations additionally are enriched in signaling cascades, whereas mutations associated with non-cancer diseases are enriched in metabolic and transport proteins.

The top 20 GO terms enriched in the disease-associated data sets tend to describe similar biological processes (Table 3). Enrichment is calculated analogously to pathway enrichment for each GO term. Cancer-associated nsSNVs are enriched in GO terms related to cell proliferation, stress response, DNA repair, transcription regulation, signal transduction or protein maturation. Somatic mutations appear more frequently in proteins related to signal transduction and not so often to DNA repair and stress response. In contrast, mutations related to non-cancer diseases are associated with metabolic processes and transmembrane transport.

Vogelstein *et al.*^30^ have recently presented a brilliant analysis of pathways accounting for major cancer driver genes and selected 12 key pathways, which largely overlap with the lists above. Vogelstein *et al.*^30^ point out that mutations in growth factor-related signaling pathways often enable cells to proliferate in conditions of limited nutrient concentration, typical for tumors. On the other hand, mutations in proteins controlling DNA damage are also frequently observed among cancer drivers, which allows to acquire secondary mutations with an increased rate. We observe that proteins of regulatory cascades are enriched with somatic, whereas proteins involved in DNA repair more frequently harbor germline mutations.

### Prediction of mechanisms of disease-associated mutations

Cancer-related malignancy often progresses through gain or loss of function of particular genes. However, it has recently been shown that alteration of gene activity can contribute to cancer progress in at least 5% of all cases.^56^ PMD (Protein Mutant Database)^57^ is a literature-based collection of data on how mutations in proteins alter their activity. For 821 disease-associated nsSNV also described in PMD, we were able to find a mapping into a potentially homologous 3D structure in this study. Of the 821 disease-associated nsSNVs described in PMD, 63 are germline cancer-associated, 107 are somatic cancer-associated and 651 are associated with non-cancer diseases. In contrast, only nine common nsSNVs and one benign nsSNV are annotated as altering protein function in PMD. This is in agreement with the expectation that common and benign variants do not have significant impact on phenotype.

A simple example of functional prediction produced by our method is the case of VHL (Von Hippel-Lindau disease tumor suppressor) gene. This gene harbors many mutations that lead to cancer, either somatic or germline (Leu155Pro, Cys162Phe, Arg167Gln, Leu188Val), which can be mapped to interaction interface with transcription elongation factor EloC (PDB id 4AJY). Other cancer-associated mutations (Asn78Ser, Tyr98His) are found on the interaction interface with a peptide of Hypoxia-inducible factor 1-alpha (HIF1alpha). Experimental data suggest that these mutations abolish the ability of VHL to bind EloC and regulate HIF1alpha.^58, 59^

In many cases, altered affinity to natural substrates and inhibitors can be explained by direct interaction with the corresponding low molecular-weight ligand. A well-known example of altered enzyme specificity is IDH1, in which mutating Arg132 changes the reaction from converting isocitrate to alpha-ketoglutarate to converting alpha-ketoglutarate to R(−)-2-hydroxyglutarate, which leads to glioma.^60^ This residue is 3.18 Å apart from alpha-ketoglutarate (PDB id 4L06). In other cases, structural analysis explains resistance to inhibitors. For example, decreased affinity of Abl1 to imatinib upon mutations Thr315Ile, Tyr253His^61^ can be explained by a tight contact with imatinib analog in a 3D structure of a complex (PDB id 2G1T). In HRas, an oncogenic mutation Gly12Asp that hinder formation of the transition state complex with a GTPase-activating protein^62^ can be found in contact with GTP in a 3D structure of HRas:GTP complex (PDB id 4K81). Analogously, Ala59Thr and Gln61Leu are in contact with GTP in a complex with PDB id 2UZI.

In androgen receptor (AR), many mutations are associated with androgen insensitivity syndrome, which leads to malformation of genitalia both in male and female. Most of these mutations are found to be in contact with androgen or its analog in one of many experimentally resolved 3D structures of AR. However, Arg840Cys and Ile869Met^63^ are distant from the ligand in these structures, and only homologous residues Gly698 and Glu727 can be found in contact with a larger prodrug ligand in glucocorticoid receptor (PDB id 4UDD, sequence identity 50%). These contacts may represent alternative interactions in a different conformation, for example, during ligand binding or dissociation.

Experimentally resolved 3D structures of oncogenes may also fail to explain specific modes of action of mutations. For example, in Ret tyrosine kinase, the Leu790Phe germline mutation is located in the vicinity of the inhibitor binding pocket. In 3D structures of Ret co-crystallized with various inhibitors, the distances between the mutated residue and the inhibitor are at least 5.88 Å. However, in a 3D structure of a homologous tyrosine kinase SYK with an inhibitor (PDB id 3TUC), distance between the corresponding residue Met435 and the inhibitor is 3.58 Å. In other cases one needs to consider homologous complexes. The complex of cyclin-dependent kinase CDK4 with its inhibitor D has not been experimentally resolved, CDK4 inhibitor D has been co-crystallized with CKD6 (PDB id 1BI8), which shares 65% identity with CDK4. In this structure, a germline mutation Arg24His in CDK4 corresponds to Arg31, which is only 2.73 Å away from the contacting protein.

Mutations in DNA-binding proteins can often be mapped to protein–DNA interaction interfaces, in many cases with the help of 3D structures of homologs. We observe homology over large evolutionary distances, due to high conservation of typical DNA-binding folds. For example, in homeobox protein HESX-1 a mutation Arg160Cys is associated with septooptic dysplasia and phenotypically results in the loss of DNA binding.^64^ A complex of HESX-1 with DNA is not experimentally resolved, but HESX-1 sequence is 52% identical to homeobox protein aristaless from *Drosophila melanogaster* that is also involved in morphogenesis. In a complex of aristaless with DNA (PDB id 3LNQ) the corresponding residue Arg137 is located 2.80 Å apart from DNA interacting with a backbone sugar in the major groove. In another case, in pituitary-specific positive transcription factor 1, which also does not have an experimentally resolved 3D structure, a mutation Glu174Gly is involved in combined pituitary hormone deficiency. It can be mapped into a complex of the rat ortholog Pou1f1 (84% identity) with DNA, where the corresponding Glu174 is 4.48 Å away from the DNA.

Another interesting case is presented by Arg882His mutation in DNMT3A, a DNA methyltransferase, which is associated with acute myeloid leukemia, and has been shown to impair formation of active homotetramers by forming stable inactive heterodimers that involve one wild-type and one mutated subunit.^65^ The mutation is annotated as potential intersubunit contact in our analysis based on a 3D structure of DNMT3A-DNMT3L complex with histone H3 (PDB id 4U7T). Hence, function of Arg882His in formation of homotereamers is unclear from these data. However, structural superimposition of this complex with HhaI-DNA complex (PDB id 1MHT), which lacks detectable sequence similarity between the structurally similar subunits, places this mutation on the DNA-binding interface as well. This presents an alternative scenario, in which Arg882His interferes with DNA binding and thus renders the heterodimer complex inactive. This latter observation also suggests an extension of our procedure, in which not only sequence homology, but also structural similarity can be taken into account.

## Discussion

Using the recently developed tool StructMAn,^28^ we have analyzed the spatial distribution of pathogenic nsSNVs in protein 3D structures. Particularly, we have considered cancer-associated nsSNVs, distinguishing between germline and somatic mutations, nsSNVs associated with non-cancer diseases, as well as common and benign variants. We considered their location with respect to protein surface or core, and various interacting molecules, namely other proteins, low molecular-weight ligands, and DNA. Taking into account 3D structures of homologous proteins considerably expands the spectrum of mutations amenable for this analysis, whereas preserving the qualitative characteristics of the structural data set. This provides a statistically powerful overview of the trends of disease-associated nsSNVs to be spatially localized to certain protein regions.

Cancer-associated nsSNVs tend to be enriched in protein–protein interaction interfaces as has been previously shown for different sets of cancer-associated mutations.^40, 41^ However, we show that random mutations in the same proteins exhibit the same pattern. We hypothesized that this enrichment can be explained by the properties of the harbouring proteins that tend to have more PPI interfaces and act as hubs of PPI networks.^48^ However, correction for the bias introduced by the number of available templates for a nsSNV suggests that this is not the case either, in line with previously observed artifacts in protein–protein interaction networks.^49^ Thus, our analysis does not confirm that disease-associated nsSNVs are specifically targeted to protein–protein interaction interfaces.

We show that cancer-associated nsSNVs, although enriched in DNA-binding proteins, are rarely located in the DNA-binding interface itself, and thus likely do not disrupt, but rather modulate interaction with DNA or the stability of the DNA-binding domain. In contrast, nsSNVs associated with non-cancer diseases, in those relatively rare cases when they occur in DNA-binding proteins, tend to localize in DNA-binding interfaces directly. NsSNVs associated with non-cancer diseases are also enriched in protein core, where they probably affect protein overall stability. The common variants are significantly depleted from all interaction interfaces, as shown by comparison with randomly selected amino-acid residues in the same proteins. Among cancer-associated nsSNVs there is a significant difference between mutations located in oncogenes and TSG: the former tend to localize in protein-ligand-binding sites, whereas the latter do not have a tendency to be located in these sites.

To put our analysis into the wider context of the research field, we compared our structural annotations to predictions of various tools that assess functional impact of genetic variants. Annovar^66^ is a convenient software framework implementing many such tools, including SIFT,^11^ PolyPhen-2,^12^ FATHMM,^67^ LRT,^68^ MutationTaster,^69^ MutationAssessor,^70^ PROVEAN,^71^ MetaSVM^72^ and MetaLR.^73^ These tools provide a classification of variants into two or more classes, of which some correspond to potentially deleterious variants and some are benign. We have applied Annovar to nsSNVs in the original non-randomized data sets and compared structural classes to functional annotations (Supplementary Table S6). We were able to collect enough annotations to allow for statistical analysis only for somatic cancer-associated and for common variants. For all annotation tools, cancer-associated nsSNVs are annotated as deleterious significantly more often than common variants (*P*-value in Fisher's exact test <2.2e-16).

In addition, we compared annotations within different structural classes. In most cases protein, ligand and DNA contacts, as well as core nsSNVs, are enriched with potentially damaging variants (Supplementary Table S6). This enrichment is statistically significant (*P*-value in Fisher's exact test <0.05) in all but one cases among cancer-associated somatic nsSNVs. In a similar comparison for common variants, contacts and core mutations are significantly enriched with predicted deleterious variants in 32 of 44 cases. Thus, we show that our structural annotations not only agree well with predictions of other common tools, but also extend them by suggesting a mechanistic explanation of the observed phenotype for with disease-associated nsSNVs.

To our knowledge, this study is the most comprehensive to date in an emerging field at the confluence of oncogenomics and structural bioinformatics. Structural comparison presented here provides a new angle at the long-standing problem of the functional characterizations of genetic variations.

## Materials and methods

### Data sets of nsSNVs

We used the Uniprot,^74^ ClinVar^3^ and COSMIC^42^ databases to construct the data sets of disease-associated mutations used in this study. In Uniprot, human disease mutations are listed in the 'humsavar.txt' file (ftp.uniprot.org). Cancer-related mutations are selected based on specific keywords in the field describing effects of natural variants or known cancer syndromes in disease acronyms. Variant effect description and origin for cancer variants (somatic or germline) have been parsed from UniProt XML file 'uniprot_sprot.xml.gz'. For ClinVar, 'Pathogenic' or 'Likely pathogenic' (but not 'Benign') nsSNVs were extracted and separated into cancer-related and non-cancer disease mutations using the NCBI MedGen disease classification. Cancer variants were classified as somatic or germline based on the 'Origin' field in ClinVar. From all the variants found in COSMIC Cancer Census genes, another source of somatic cancer missense mutations, we included only variants observed in at least two cancer samples in order to enrich them with potentially functional 'drivers'. Gene information (oncogene or suppressor and so on) was extracted from the 'cancer_gene_census.csv' file. Common missense variants were selected from ExAC 0.3.1^43^ based on the condition that alternative allele frequency is ⩾5% in at least one ExAC population. We assume these variants are common, and thus not associated with any detrimental phenotype. As another set of functionally neutral variants, we constructed a set of nsSNVs from ClinVar that are annotated as 'Benign'. All nsSNVs present in a neutral data set and a disease-associated data set were removed from the disease-associated data set (we observed only 151 such cases). In addition, we have considered ClinVar nsSNVs with different review status, and found no difference in the spatial distribution of such variants, provided that the size of the subset is comparable with the size of the original set (Supplementary Figure S6).

We created a randomized control data set corresponding to each of the disease-associated and neutral sets described above by taking the genes from these data sets and introducing the same number of nsSNVs in them at random positions in the nucleotide sequence. These data sets represent artificial random mutations without considering natural repair mechanisms or possible evolutionary consequences of potential damage introduced by the mutations, and therefore are likely to exhibit a different spatial distribution from the biological data sets. The resulting non-redundant and non-overlapping data sets are described in the Table 1.

### Template recognition

In order to perform structural annotation of entire mutation data sets, we search for all available 3D structures of proteins from these sets, as well as of their homologs (templates) with the StructMAn pipeline.^28^ This tool takes a list of gene identifiers and amino-acid replacements as input. Then for each gene, its amino-acid sequence from UniProt^74^ is searched against all proteins with resolved 3D structures from the PDB^75^ with BLAST^76^ (e-value <10^−5^, alignment coverage >50% of shorter protein length or longer than 50 amino-acid residues, sequence identity > 35%). The sequence of the correct isoform is always used when this information is available. In other cases, the canonical isoform as defined in UniProt is used. nsSNVs are mapped onto the sequences of the templates using a global pairwise alignment algorithm from the EMBOSS package.^77^ If a mutation is mapped to a gap, the corresponding template is discarded. For each PDB entry from the resulting list of templates, a template score is calculated based on the following four attributes: (1) sequence identity, (2) alignment coverage, (3) resolution and (4) R factor.^28^ When multiple templates were available for a single nsSNV, all templates were considered to ensure its fullest possible structural annotation.

### Structural annotations of nsSNVs

Structural analysis has been performed for each template, even if an experimental 3D structure of the target protein is available, in order to collect as much relevant information, such as positions of macromolecular interaction partners and ligands, as possible. We compute (1) the shortest distance of the mutated residue to a ligand, (2) the shortest distance to any other macromolecule and (3) the relative surface accessibility of the substituted residue using NACCESS.^78^ A combination of these factors with the corresponding template score produces the final candidate score, which reflects the similarity between the sequences of the template structure and the corresponding human protein, the structural quality of the template, as well as the propensity of the mutated amino-acid residue to be located in a potentially functionally important region of the structure:



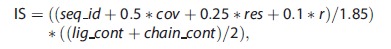



where IS is the interaction score, *seq_id* is sequence identity between template and target, *cov* is the target coverage by the alignment to the template, *res=*1/(1+exp(1.5*resolution–4)), *r* is 1−R-value, *lig_cont*=1/(1+exp(*SLD*−10)), *chain_cont*=1/(1+exp(*SCD*−10)), *SLD* is the shortest distance between the substituted residue and a ligand, *SCD* is the shortest distance between the substituted residue and any other macromolecule. For more details, see.^28^

### Pathway and GO-term enrichment analysis

To facilitate the pathway and GO-term enrichment analysis, we constructed a combined protein-level score as the maximal sum over all templates of the candidate scores for all mutations in that protein normalized such that proteins containing a small number of high-scoring candidate mutations receive a higher combined score than proteins containing multiple low-scoring candidate mutations (see more details in reference 28). All protein scores corresponding to a certain pathway in the Reactome Pathway Database^55^ or to a certain GO term were summed, normalized by the number of corresponding proteins, and compared between disease-associated data sets and sets of neutral variants. For each pathway and GO term overlapping between a disease-associated set and a set of neutral variants, we subtract the two corresponding scores to obtain differential scores. High differential scores indicate enrichment in nsSNVs with probably high impact on protein interactions for this pathway or GO term.

Top 20 pathways and GO terms for each comparative analysis are given in Tables 2 and 3. We performed the same comparative analysis using the randomized data sets instead of the set of common variants. These randomized sets were created by choosing 10 000 random human genes and inserting in them a number of mutations sampled from the distribution of the number of nsSNVs per gene for all biological data sets. The number of nsSNVs per gene was not found to correlate with gene length in the biological data, so we did not account to gene length in this sampling. We found the results of the pathway and GO-term enrichment analysis in this setting to be highly similar to the data obtained in comparison with common nsSNVs (Supplementary Table S7). This indicates, that the pathways and GO terms enriched in structurally impactful nsSNVs are not an artifact of the skewed distribution of proteins in the input data sets.

### Definition of structural classes and chemical properties of mutated residues

We have also defined five non-overlapping structural classes: 'Surface', 'Core', 'DNA contacts', 'Ligand contacts' and 'Protein contacts'. To assign an nsSNV to a structural class, we first calculated distances to the nearest protein and DNA chains and to low molecular-weight ligands. To define low molecular-weight ligands, we considered all HETATM records in PDB files, excluding modified amino-acid residues, and manually removed all common crystallographic buffer components. Metal ions were kept, as they often play an important functional role, and mutations affecting their binding may have a severe effect. The shortest distance among distances to all interaction partners was chosen, and if it was lower than 5 Å, the nsSNV was assigned to the corresponding contact structural class. If no contacting molecule was found within 5 Å, we calculated the solvent accessible area of the corresponding amino-acid residue with NACCESS.^78^ If it was below 16%, the nsSNV was assigned to the class 'Core',^79^ otherwise the nsSNV was assigned to the class 'Surface'.

To measure the difference of chemical properties for each pair of wild-type and mutated amino-acid residues, we used a vector of five numerical descriptors^80^ to represent all 237 physical–chemical properties of amino-acid residues from,^81^ and calculated Euclidean distances between the end points of these vectors. Alternatively, we calculated chemical distances as corresponding values of the Blosum62 substitution matrix.^82^ When multiple mutations were observed for a position, we computed the average over all observed mutations.

### PPI networks

To identify protein complexes containing mutations in different subunits we constructed PPI networks. We considered two proteins interacting, if they are mapped to subunits in at least one template structure. In order to reduce the number of false-positive complexes we allowed only templates with sequence identity above 90%. We display only mutated subunits (Supplementary Figures S3–S5), whereas the complexes may contain additional subunits without mutations. Complexes with only one mutated subunit are not shown in the network.

## Publisher’s note:

Springer Nature remains neutral with regard to jurisdictional claims in published maps and institutional affiliations.

## Figures and Tables

**Table 1:** Data sets in this study

*Data set*	*Source database*	*Variants (genes)*	*Variants (genes) with an experimentally resolved 3D structure of the corresponding protein (>98% sequence identity)*	*Variants (genes) mapped onto homologous proteins with experimentally resolved 3D structure*
Cancer germline	ClinVar, Uniprot	452 (86)	360, 79.6% (58, 67.4%)	450, 99.6% (86, 100%)
Cancer germline randomized	n/a	443±3 (86)	268±11, 60.5% (44±2, 51.2%)	318±12, 71.8% (86, 100%)
Cancer somatic	ClinVar, Uniprot, COSMIC	3673 (371)	2952, 80.4% (246, 66.3%)	3660, 99.6% (371, 100%)
Cancer somatic randomized	n/a	3572±8 (371)	1983±17, 55.5% (192±4, 51.7%)	2398±21, 67.1% (371, 100%)
Non-cancer diseases	ClinVar, Uniprot	14983 (1586)	9678, 64.6% (795, 50.1%)	14386, 96.0% (1586, 100%)
Non-cancer diseases randomized	n/a	14431±23 (1586)	7800±35, 54.0% (748±8, 47.2%)	10982±54, 76.1% (1586, 100%)
Common	ExAC	27326 (10261)	2048, 7.5% (1038, 10.1%)	6048, 22.1% (5251, 51.1%)
Common randomized	n/a	27214±11 (10261)	2425±19, 8.9% (1260±19, 12.3%)	7091±57, 26.1% (5251, 51.1%)
Benign	ClinVar	5186 (962)	658, 12.7% (208, 21.6%)	1166, 22.5% (634, 65.9%)
Benign randomized	n/a	5134±8 (962)	765±21, 14.9% (239±4, 24.8%)	1387±18, 27.0% (634, 65.9%)

**Table 2:** Top 20 ReactomeDB pathways identified in differential analysis of disease-associated data sets compared with the set of common variants

*Germline cancer-associated mutations*	*Somatic cancer-associated mutations*	*Mutations associated with non-cancer diseases*
Regulation of TP53 activity through phosphorylation	PIP3 activates AKT signaling	Neutrophil degranulation
Ub-specific processing proteases	Oxidative stress-induced senescence	Intrinsic pathway of fibrin clot formation
TP53 regulates transcription of DNA repair genes	Factors involved in megakaryocyte development and platelet production	Glycosphingolipid metabolism
G2/M DNA damage checkpoint	Oncogene-induced senescence	Gap junction assembly
Recruitment and ATM-mediated phosphorylation of repair and signaling proteins at DNA double strand breaks	Ub-specific processing proteases	Urea cycle
Factors involved in megakaryocyte development and platelet production	Ovarian tumor domain proteases	Platelet degranulation
PIP3 activates AKT signaling	Regulation of TP53 degradation	Oligomerization of connexins into connexons
Stabilization of p53	Regulation of TP53 activity through Phosphorylation	Transport of connexins along the secretory pathway
Regulation of TP53 activity through methylation	Pre-NOTCH transcription and translation	Galactose catabolism
Regulation of TP53 degradation	Recruitment and ATM-mediated phosphorylation of repair and signaling proteins at DNA double strand breaks	Transport of gamma-carboxylated protein precursors from the endoplasmic reticulum to the Golgi apparatus
Formation of senescence-associated heterochromatin foci (SAHF)	Association of TriC/CCT with target proteins during biosynthesis	Removal of aminoterminal propeptides from gamma-carboxylated proteins
Oncogene induced senescence	TP53 regulates transcription of DNA repair Genes	Gamma-carboxylation of protein precursors
Oxidative stress-induced senescence	G2/M DNA damage checkpoint	Extrinsic pathway of fibrin clot formation
DNA damage/telomere stress-induced Senescence	TP53 regulates metabolic genes	Common pathway of fibrin clot formation
SUMOylation of transcription factors	Regulation of TP53 activity through methylation	Striated muscle contraction
Activation of NOXA and translocation to mitochondria	Regulation of TP53 activity through acetylation	RAF/MAP kinase cascade
Regulation of TP53 activity through acetylation	TP53 regulates transcription of genes involved in Cytochrome C release	Regulation of gene expression in beta cells
Transcriptional activation of cell cycle inhibitor p21	Stabilization of p53	Phenylalanine and tyrosine catabolism
PI5P regulates TP53 acetylation	Regulation of TP53 activity through association with co-factors	Signaling by BRAF and RAF fusions
TP53 regulates transcription of additional cell cycle genes whose exact role in the p53 pathway remain uncertain	DNA damage/telomere stress-induced senescence	Signaling by RAS mutants

Differences of the combined scores (see Materials and methods) for disease-associated nsSNVs and common variants are shown in parentheses.

**Table 3:** GO-term enrichment analysis: top 20 terms in the 'Process' category

*Germline cancer-associated mutations*	*Somatic cancer-associated mutations*	*Mutations associated with non-cancer diseases*
Positive regulation of transcription, DNA-templated	Negative regulation of cell proliferation	Positive regulation of transcription, DNA-templated
Negative regulation of cell proliferation	Positive regulation of transcription, DNA-templated	Cell–cell signaling
Negative regulation of transcription from RNA polymerase II promoter	Negative regulation of transcription from RNA polymerase II promoter	Response to drug
Negative regulation of apoptotic process	Negative regulation of apoptotic process	Blood coagulation
Regulation of transcription, DNA-templated	Positive regulation of transcription from RNA polymerase II promoter	Positive regulation of transcription from RNA polymerase II promoter
Cell proliferation	Regulation of transcription, DNA-templated	Transport
Positive regulation of gene expression	Ras protein signal transduction	Positive regulation of gene expression
Positive regulation of transcription from RNA polymerase II promoter	Positive regulation of gene expression	Visual perception
Regulation of signal transduction by p53 class mediator	Negative regulation of transcription, DNA-templated	Signal transduction
Cellular response to DNA damage stimulus	Cell proliferation	Negative regulation of neuron apoptotic process
DNA damage response, signal transduction by p53 class mediator resulting in transcription of p21 class mediator	Negative regulation of cell growth	Negative regulation of apoptotic process
Intrinsic apoptotic signaling pathway in response to DNA damage by p53 class mediator	Cell cycle arrest	Nervous system development
Ras protein signal transduction	Viral process	Negative regulation of transcription from RNA polymerase II promoter
Regulation of apoptotic process	Cellular response to drug	Liver development
Cellular response to drug	Cellular response to DNA damage stimulus	Sensory perception of sound
Cell differentiation	Positive regulation of apoptotic process	ER to Golgi vesicle-mediated transport
Response to X-ray	Replicative senescence	Positive regulation of cell proliferation
Negative regulation of transcription, DNA-templated	Cell differentiation	Response to hypoxia
Cell cycle arrest	Regulation of apoptotic process	Response to estradiol
Negative regulation of cell growth	Regulation of signal transduction by p53 class mediator	Transcription, DNA-templated

Differences of the combined scores (see Materials and methods) for disease-associated nsSNVs and common variants are shown in parentheses.

## Supplementary Material

Supplementary Materials

Supplementary Fiugres

## References

[bib1] Cooper DN, Krawczak M, Polychronakos C, Tyler-Smith C, Kehrer-Sawatzki H. Where genotype is not predictive of phenotype: towards an understanding of the molecular basis of reduced penetrance in human in-herited disease. Hum Genet 2013; 132: 1077–1130. 23820649 10.1007/s00439-013-1331-2 PMC3778950

[bib2] 1000 Genomes Project Consortium. A map of human genome variation from population-scale sequencing. Nature 2010; 467: 1061–1073. 20981092 10.1038/nature09534 PMC3042601

[bib3] Landrum MJ, Lee JN, Bensom M, Brown G, Chao C, Chitipiralla S et al. ClinVar: public archive of interpretations of clinically relevant variants. Nucleic Acids Res 2016; 44: D862–D868. 26582918 10.1093/nar/gkv1222 PMC4702865

[bib4] Petukh M, Kucukkal TG, Alexov E. On human disease-causing amino acid variants: statistical study of sequence and structural patterns. Hum Mutat 2015; 36: 524–534. 25689729 10.1002/humu.22770 PMC4409542

[bib5] De Beer TAP, Laskowski RA, Parks SL, Sipos B, Goldman N, Thornton JM. Aminoacid changes in disease-associated variants differ radically from variants observed in the 1000 genomes project dataset. PLoS Comput Biol 2013; 9: 1–15. 10.1371/journal.pcbi.1003382 PMC3861039 24348229

[bib6] Kucukkal TG, Petukh M, Li L, Alexov E. Structural and physico-chemical effects of disease and non-disease nsSNPs on proteins. Curr Opin Struct Biol 2015; 32: 18–24. 25658850 10.1016/j.sbi.2015.01.003 PMC4511717

[bib7] Sahni N, Yi S, Taipale M, Fuxman Bass JI, Coulombe-Huntington J, Yang F et al. Widespread macromolecular interaction perturbations in human genetic disorders. Cell 2015; 161: 647–660. 25910212 10.1016/j.cell.2015.04.013 PMC4441215

[bib8] Yates CM, Sternberg MJ. The effects of non-synonymous single nucleotide polymorphisms (nsSNPs) on ProteinProtein interactions. J Mol Biol 2013; 425: 3949–3963. 23867278 10.1016/j.jmb.2013.07.012

[bib9] Stefl S, Nishi H, Petukh M, Panchenko AR, Alexov E. Molecular mechanisms of disease-causing missense mutations. J Mol Biol 2013; 425: 3919–3936. 23871686 10.1016/j.jmb.2013.07.014 PMC3796015

[bib10] Wang X, Wei X, Thijssen B, Das J, Lipkin SM, Yu H. Three-dimensional reconstruction of protein networks provides insight into human genetic disease. Nat Biotech 2012; 30: 159–164. 10.1038/nbt.2106 PMC3708476 22252508

[bib11] Ng PC, Henikoff S. SIFT: Predicting amino acid changes that affect protein function. Nucleic Acids Res 2003; 31: 3812–3814. 12824425 10.1093/nar/gkg509 PMC168916

[bib12] Adzhubei IA, Schmidt S, Peshkin L, Ramensky VE, Gerasimova A, Bork P et al. A method and server for predicting damaging missense mutations. Nat Methods 7: 248–249. 20354512 10.1038/nmeth0410-248 PMC2855889

[bib13] Thomas PD, Campbell MJ, Kejariwal A, Mi H, Karlak B, Daverman R et al. PANTHER: a library of protein families and subfamilies indexed by function. Genome Res 2003; 13: 2129–2141. 12952881 10.1101/gr.772403 PMC403709

[bib14] Yue P, Melamud E, Moult J. SNPs3D: candidate gene and SNP selection for association studies. BMC Bioinformatics 2006; 7: 166. 16551372 10.1186/1471-2105-7-166 PMC1435944

[bib15] Katsonis P, Lichtarge O. A formal perturbation equation between genotype and phenotype determines the Evolutionary Action of protein-coding variations on fitness. Genome Res 2014; 24: 2050–2058. 25217195 10.1101/gr.176214.114 PMC4248321

[bib16] Bromberg Y, Rost B. SNAP: predict effect of non-synonymous polymorphisms on function. Nucleic Acids Res 2007; 35: 3823–3835. 17526529 10.1093/nar/gkm238 PMC1920242

[bib17] Thusberg J, Olatubosun A, Vihinen M. Performance of mutation pathogenicity prediction methods on missense variants. Hum Mutat 2011; 32: 358–368. 21412949 10.1002/humu.21445

[bib18] De Baets G, Van Durme J, Reumers J, Maurer-Stroh S, Vanhee P, Dopazo J et al. SNPeffect 4.0: on-line prediction of molecular and structural effects of protein-coding variants. Nucleic Acids Res 2012; 40: D935–D939. 22075996 10.1093/nar/gkr996 PMC3245173

[bib19] Parthiban V, Gromiha MM, Schomburg D. CUPSAT: prediction of protein stability upon point mutations. Nucleic Acids Res 2006; 34: W239–W242. 16845001 10.1093/nar/gkl190 PMC1538884

[bib20] Yin S, Ding F, Dokholyan NV. Eris: an automated estimator of protein stability. Nat Methods 2007; 4: 466–467. 17538626 10.1038/nmeth0607-466

[bib21] Schymkowitz J, Borg J, Stricher F, Nys R, Rousseau F, Serrano L. The FoldX web server: an online force field. Nucleic Acids Res 2005; 33: W382–W388. 15980494 10.1093/nar/gki387 PMC1160148

[bib22] Zhou H, Zhou Y. Distance-scaled, finite ideal-gas reference state improves structure-derived potentials of mean force for structure selection and stability prediction. Protein Sci 2002; 11: 2714–2726. 12381853 10.1110/ps.0217002 PMC2373736

[bib23] Gilis D, Rooman M. PoPMuSiC, an algorithm for predicting protein mutant stability changes. Application to prion proteins. Protein Eng 2000; 13: 849–856. 11239084 10.1093/protein/13.12.849

[bib24] Savojardo C, Fariselli P, Martelli PL, Casadio R. INPS-MD: a web server to predict stability of protein variants from sequence and structure. Bioinformatics 2016; 32: 2542–2544. 27153629 10.1093/bioinformatics/btw192

[bib25] Grimm DG, Azencott C-A, Aicheler F, Gieraths U, MacArthur DG, Samocha KE et al. The evaluation of tools used to predict the impact of missense variants is hindered by two types of circularity. Hum Mutat 2015; 36: 513–523. 25684150 10.1002/humu.22768 PMC4409520

[bib26] Mosca R, Tenorio-Laranga J, Olivella R, Alcalde V, Céol A, Soler-López M et al. dSysMap: exploring the edgetic role of disease mutations. Nat Methods 2015; 12: 167–168. 25719824 10.1038/nmeth.3289

[bib27] Betts MJ, Lu Q, Jiang Y, Drusko A, Wichmann O, Utz M et al. Mechismo: predicting the mechanistic impact of mutations and modifications on molecular interactions. Nucleic Acids Res 2015; 43: e10. 25392414 10.1093/nar/gku1094 PMC4333368

[bib28] Gress A, Ramensky VE, Buech J, Keller A, Kalinina OV. StructMAn: annotation of single-nucleotide polymorphisms in the structural context. Nucleic Acids Res 2016; 44: W463–W468. 27150811 10.1093/nar/gkw364 PMC4987916

[bib29] Lugo-Martinez J, Pejaver V, Pagel KA, Jain S, Mort M, Cooper DN et al. The loss and gain of functional amino acid residues is a common mechanism causing human inherited disease. PLoS Comput Biol 2016; 12: 1–23. 10.1371/journal.pcbi.1005091 PMC5001644 27564311

[bib30] Vogelstein B, Papadopoulos N, Velculescu VE, Zhou S, Diaz LA, Kinzler KW. Cancer genome landscapes. Science 2013; 339: 1546–1558. 23539594 10.1126/science.1235122 PMC3749880

[bib31] Tian R, Basu MK, Capriotti E. Computational methods and resources for the interpretation of genomic variants in cancer. BMC Genomics 2015; 16: 1–19. 26111056 10.1186/1471-2164-16-S8-S7 PMC4480958

[bib32] Gnad F, Baucom A, Mukhyala K, Manning G, Zhang Z. Assessment of computational methods for predicting the effects of missense mutations in human cancers. BMC Genomics 2013; 14: 1–13. 23819521 10.1186/1471-2164-14-S3-S7 PMC3665581

[bib33] Pal LR, Moult J. Genetic basis of common human disease: insight into the role of missense snps from genome-wide association studies. J Mol Biol 2015; 427: 2271–2289. 25937569 10.1016/j.jmb.2015.04.014 PMC4893807

[bib34] Capriotti E, Altman RB, Bromberg Y. Collective judgment predicts disease-associated single nucleotide variants. BMC Genomics 2013; 14: S2. 10.1186/1471-2164-14-S3-S2 PMC3839641 23819846

[bib35] Lu H-C, Herrera Braga J, Fraternali F. PinSnps: structural and functional analysis of SNPs in the context of protein interaction networks. Bioinformatics 2016; 32: 2534–2536. 27153707 10.1093/bioinformatics/btw153 PMC4978923

[bib36] Liu X, Wu C, Li C, Boerwinkle E. dbNSFP v3.0: a one-stop database of functional predictions and annotations for human nonsynonymous and splice-site SNVs. Hum Mutat 2016; 37: 235–241. 26555599 10.1002/humu.22932 PMC4752381

[bib37] Porta-Pardo E, Hrabe T, Godzik A. Cancer3D: understanding cancer mutations through protein structures. Nucleic Acids Res 2015; 43: D968–D973. 25392415 10.1093/nar/gku1140 PMC4383948

[bib38] Weinstein JN, Collisson EA, Mills GB, Shaw KRM, Ozenberger BA, Ellrott K et al. The Cancer Genome Atlas Pan-Cancer analysis project. Nat Genet 2013; 45: 1113–1120. 24071849 10.1038/ng.2764 PMC3919969

[bib39] Barretina J, Caponigro G, Stransky N, Venkatesan K, Margolin AA, Kim S et al. The Cancer Cell Line Encyclopedia enables predictive modelling of anticancer drug sensitivity. Nature 2012; 483: 603–607. 22460905 10.1038/nature11003 PMC3320027

[bib40] Engin HB, Kreisberg JF, Carter H. Structure-Based Analysis Reveals Cancer Missense Mutations Target Protein Interaction Interfaces. PLoS ONE 2016; 11: 1–21. 10.1371/journal.pone.0152929 PMC4820104 27043210

[bib41] Kamburov A, Lawrence MS, Polak P, Leshchiner I, Lage K, Golub TR et al. Comprehensive assessment of cancer missense mutation clustering in protein structures. Proc Natl Acad Sci 2015; 112: E5486–E5495. 26392535 10.1073/pnas.1516373112 PMC4603469

[bib42] Forbes SA, Beare D, Gunasekaran P, Leung K, Bindal N, Boutselakis H et al. COSMIC: exploring the world’s knowledge of somatic mutations in human cancer. Nucleic Acids Res 2015; 43: D805–D811. 25355519 10.1093/nar/gku1075 PMC4383913

[bib43] Exome Aggregation Consortium. Analysis of protein-coding genetic variation in 60,706 humans. Nature 2016; 536: 285–291. 27535533 10.1038/nature19057 PMC5018207

[bib44] Aloy P, Ceulemans H, Stark A, Russell RB. The relationship between sequence and interaction divergence in proteins. J Mol Biol 2003; 332: 989–998. 14499603 10.1016/j.jmb.2003.07.006

[bib45] Nagy R, Sweet K, Eng C. Highly penetrant hereditary cancer syndromes. Oncogene 2004; 23: 6445–6470. 15322516 10.1038/sj.onc.1207714

[bib46] Dosztányi Z, Csizmók V, Tompa P, Simon IJ. The pairwise energy content estimated from amino acid composition discriminates between folded and intrinsically unstructured proteins. J Mol Biol 2005; 347: 827–839. 15769473 10.1016/j.jmb.2005.01.071

[bib47] Touw WG, Baakman C, Black J, te Beek TAH, Krieger E, Joosten RP et al. A series of PDB related databases for everyday needs. Nucleic Acids Res 2015; 43: D364–D368. 25352545 10.1093/nar/gku1028 PMC4383885

[bib48] Goh K, Cusick ME, Valle D, Childs B, Vidal M, Barabási A-L. The human disease network. Proc Natl Acad Sci 2007; 104: 8685–8690. 17502601 10.1073/pnas.0701361104 PMC1885563

[bib49] Schaefer MH, Serrano L, Andrade-Navarro MA. Correcting for the study bias associated with protein–protein interaction measurements reveals differences between protein degree distributions from different cancer types. Front Genet 2015; 6: 260. 26300911 10.3389/fgene.2015.00260 PMC4523822

[bib50] Bullock AN, Henckel J, DeDecker BS, Johnson CM, Nikolova PV, Proctor MR et al. Thermodynamic stability of wild-type and mutant p53 coredomain. Proc Natl Acad Sci 1997; 94: 14338–14342. 9405613 10.1073/pnas.94.26.14338 PMC24967

[bib51] Joerger AC, Fersht AR. Structure-function-rescue: the diverse nature of common p53 cancer mutants. Oncogene 2007; 26: 2226–2242. 17401432 10.1038/sj.onc.1210291

[bib52] Guerrero-Preston R, Michailidi C, Marchionni L, Pickering CR, Frederick MJ, Myers JN et al. Key tumor suppressor genes inactivated by ‘greater promoter’ methylation and somatic mutations in head and neck cancer. Epigenetics 2014; 9: 1031–1046. 24786473 10.4161/epi.29025 PMC4143405

[bib53] Law V, Knox C, Djoumbou Y, Jewison T, Guo AC, Liu Y et al. DrugBank 4.0: shedding new light on drug metabolism. Nucleic Acids Res 2014; 42: D1091–D1907. 24203711 10.1093/nar/gkt1068 PMC3965102

[bib54] Baselga J. Targeting tyrosine kinases in cancer: the second wave. Science 2006; 312: 1175–1178. 16728632 10.1126/science.1125951

[bib55] Croft D, Mundo AF, Haw R, Milacic M, Weiser J, Wu G et al. The Reactome pathway knowledgebase. Nucleic Acids Res 2014; 42: D472–D477. 24243840 10.1093/nar/gkt1102 PMC3965010

[bib56] Reva B, Antipin Y, Sander C. Predicting the functional impact of protein mutations: application to cancer genomics. Nucleic Acids Res 2011; 39: e118. 21727090 10.1093/nar/gkr407 PMC3177186

[bib57] Kawabata T, Ota M, Nishikawa K. The protein mutant database. Nucleic Acids Res 1999; 27: 355–357. 9847227 10.1093/nar/27.1.355 PMC148182

[bib58] Clifford SC, Cockman ME, Smallwood AC, Mole DR, Woodward ER, Maxwell PH et al. Contrasting effects on HIF-1 regulation by disease-causing pVHL mutations correlate with patterns of tumourigenesis in von Hippel-Lindau disease. Hum Mol Genet 2001; 10: 1029–1038. 11331613 10.1093/hmg/10.10.1029

[bib59] Hoffman MA, Ohh M, Yang H, Klco JM, Ivan M, Kaelin WG Jr. von Hippel-Lindau protein mutants linked to type 2C VHL disease preserve the ability to downregulate HIF. Hum Mol Genet 2001; 10: 1019–1027. 11331612 10.1093/hmg/10.10.1019

[bib60] Dang L, White DW, Gross S, Gennet BD, Bittinger MA, Fantin VR et al. Cancer-associated IDH1 mutations produce 2-hydroxyglutarate. Nature 2009; 465: 966. 10.1038/nature09132 PMC3766976 20559394

[bib61] Azam M, Latek RR, Daley GQ. Mechanisms of autoinhibition and STI-571/imatinib resistance revealed by mutagenesis of BCR-ABL. Cell 2003; 112: 831–843. 12654249 10.1016/s0092-8674(03)00190-9

[bib62] Gremer L, Gilsbach B, Reza Ahmadian M, Wittinghofer A. Fluoride complexes of oncogenic Ras mutants to study the Ras-RasGAP interaction. Biol Chem 2008; 389: 1163–1171. 18713003 10.1515/BC.2008.132

[bib63] Bevan CL, Brown BB, Davies HR, Evans BAJ, Hughes IA, Patterson MN. Functional analysis of six androgen receptor mutations identified in patients with partial androgen insensitivity syndrome. Hum Mol Genet 1996; 5: 265–273. 8824883 10.1093/hmg/5.2.265

[bib64] Brickman JM, Clements M, Tyrell R, McNay D, Woods K, Warner J et al. Molecular effects of novel mutations in Hesx1/HESX1 associated with human pituitary disorders. Development 2001; 128: 5189–5199. 11748154 10.1242/dev.128.24.5189

[bib65] Russler-Germain DA, Spencer DH, Young MA, Lamprecht TL, Miller CA, Fulton R et al. The R882H DNMT3A mutation associated with AML dominantly inhibits wild-type DNMT3A by blocking its ability to form active tetramers. Cancer Cell 2014; 25: 442–454. 24656771 10.1016/j.ccr.2014.02.010 PMC4018976

[bib66] Wang K, Li M, Hakonarson H. ANNOVAR: Functional annotation of genetic variants from next-generation sequencing data. Nucleic Acids Res 2010; 38: e164. 20601685 10.1093/nar/gkq603 PMC2938201

[bib67] Shihab HA, Gough J, Cooper DN, Stenson PD, Barker GLA, Edwards KJ, Day INM et al. Predicting the functional, molecular and phenotypic consequences of amino acid substitutions using hidden markov models. Hum Mutat 2013; 34: 57–65. 23033316 10.1002/humu.22225 PMC3558800

[bib68] Chun S, Fay JC. Identification of deleterious mutations within three human genomes. Genome Res 2009; 19: 1553–1561. 19602639 10.1101/gr.092619.109 PMC2752137

[bib69] Schwarz JM, Cooper DN, Schuelke M, Seelow D. MutationTaster2: mutation prediction for the deep-sequencing age. Nat Methods 2014; 11: 361–362. 24681721 10.1038/nmeth.2890

[bib70] Reva B, Antipin Y, Sander C. Predicting the functional impact of protein mutations: application to cancer genomics. Nucleic Acids Res 2011; 39: e118. 21727090 10.1093/nar/gkr407 PMC3177186

[bib71] Choi Y, Chan AP. PROVEAN web server: a tool to predict the functional effect of amino acid substitutions and indels. Bioinformatics 2015; 31: 2745–2747. 25851949 10.1093/bioinformatics/btv195 PMC4528627

[bib72] SungHwan K, Jae-Hwan J, JungJun L, Ja-Yong K. Meta-analytic support vector machine for integrating multiple omics data. BioData Min 2017; 10: 2. 28149325 10.1186/s13040-017-0126-8 PMC5270233

[bib73] Dong C, Wei P, Jian X, Gibbs R, Boerwinkle E, Wang K et al. Comparison and integration of deleteriousness prediction methods for nonsynonymous SNVs in whole exome sequencing studies. Hum Mol Genet 2015; 24: 2125–2137. 25552646 10.1093/hmg/ddu733 PMC4375422

[bib74] UniProt Consortium. UniProt: a hub for protein information. Nucleic Acids Res 43: D204–D212. 25348405 10.1093/nar/gku989 PMC4384041

[bib75] Berman HM, Westbrook J, Feng Z, Gilliland G, Bhat TN, Weissig H et al. The protein data bank. Nucleic Acids Res 2000; 28: 235–242. 10592235 10.1093/nar/28.1.235 PMC102472

[bib76] Altschul SF, Gish W, Miller W, Myers EW, Lipman DJ. Basic local alignment search tool. J Mol Biol 1990; 215: 403–410. 2231712 10.1016/S0022-2836(05)80360-2

[bib77] Rice P, Longden I, Bleasby A. EMBOSS: the european molecular biology open software suite. Trends Genet 2000; 16: 276–277. 10827456 10.1016/s0168-9525(00)02024-2

[bib78] Hubbard S, Thornton J. Naccess V2.1.1solvent accessible area calculations. Available at http://www.bioinf.manchester.ac.uk/naccess/nac_intro.html. 1992.

[bib79] Rost B, Sander C. Conservation and prediction of solvent accessibility in protein families. Proteins 1994; 20: 216–226. 7892171 10.1002/prot.340200303

[bib80] Venkatarajan SM, Braun W. New quantitative descriptors of amino acids based on multidimensional scaling of a large number of physical–chemical properties. Mol Model Annu 2001; 7: 445–453.

[bib81] Kawashima S, Ogata H, Kanehisa M. AAindex: amino acid index database. Nucleic Acids Res 1999; 27: 368–369. 9847231 10.1093/nar/27.1.368 PMC148186

[bib82] Henikoff S, Henikoff JG. Amino acid substitution matrices from protein blocks. Proc Natl Acad Sci USA 1992; 89: 10915–10919. 1438297 10.1073/pnas.89.22.10915 PMC50453

