# Supplementary material for: Spatial distribution of disease-associated variants in three-dimensional structures of protein complexes
Source: Oncogenesis. 2017 Sep 25;6(9):e380–. doi: 10.1038/oncsis.2017.79 (PMC5623905; doi:10.1038/oncsis.2017.79)
Supplement: Supplementary Materials [file oncsis201779x1.docx]

**Supplementary materials**

**Supplementary Figure S1. A.** Chemical distance between wildtype and mutated residues for the subset of templates with sequence identity greater or equal 90%. Chemical distance is calculated as Euclidean distances between the end points of the vectors representing five most important numerical descriptors of physical and chemical properties [80] of the wildtype and mutant amino acids. **B and C**. Similarity between wild-type and mutant residues according to the BLOSUM62 substitution matrix for the full set of templates (B) and for the subset of templates with sequence identity greater or equal 90% (C).

**Supplementary Figure S2.** Venn diagrams representing distribution of amino acids corresponding to nsSNVs from different disease-associated and neutral datasets. The criterion of belonging to a particular contact class is the distance to an interaction partner < 5 Å in at least one template structure.

**Supplementary Figure S3.** Network representing all protein complexes with germline cancer-associated mutation in different subunits. Self edges correspond to homooligomers.

**Supplementary Figure S4.** Network representing all protein complexes with somatic cancer-associated mutation in different subunits. Self edges correspond to homooligomers.

**Supplementary Figure S5.** Network representing all protein complexes with mutation associated with non-cancer diseases in different subunits. Self edges correspond to homooligomers.

**Supplementary Figure S6.** Structural classes in sets of nsSNVs with different review status in ClinVar and the corresponding general biological datasets. Total numbers of annotated nsSNVs are given in parentheses in the labels of the respective bars.

**Supplementary Table S1.** Distribution of nsSNVs in different secondary structure elements. Secondary structure was assigned by running DSSP [50] for all template structures and a majority vote over the assignments. DSSP annotations 'H', 'G', 'I' were combined into 'helix' class, 'E' is represented as 'sheet', other annotations as 'disorder'. For randomized datasets, cumulative numbers are given.

**Supplementary Table S2.** Distribution of nsSNVs over mutually-exclusive contact classes (see text for details). Mean values and standard deviations are given for 1000 bootstrap runs for the biological datasets and over 10 replicas for randomized datasets. P-values were calculated in Fisher's exact test, mean values were used for randomized datasets. Significance of differences between the distributions of distances from mutations corresponding to nsSNVs to the closest interaction partners. P-values in Wilcoxon two-sided tests are given.

**Supplementary Table S3.** Distribution of structural classes in disease-associated datasets compared to 100 randomly sampled equally sized sets from the sets of common and benign variants with the same distribution of identified template structures as in the corresponding disease-associated datasets. Insignificant differences (within four standard deviations) are marked in bold red.

**Supplementary Table S4.** All structural annotations for all disease-associated and neutral nsSNVs. Available templates, minimal distances to interaction partners, assignment to structural classes, secondary structure assignment, and functional annotations using Annovar [54] according to documentation at<http://annovar.openbioinformatics.org/en/latest/user-guide/filter/> are provided.

**Supplementary Table S5.** All structural annotations for mutations in oncogenes and TSGs. The same data as in Supplementary Table S4 is provided. Distribution of nsSNVs over mutually-exclusive contact classes for oncogenes and TSGs. Mean values and standard deviations are given for 1000 bootstrap runs. P-values in Wilcoxon two-sided test between distributions to the nearest interaction partner across oncogenes, TSGs, and the general sets of germline and somatic nsSNVs. Distribution of structural classes in the sets of nsSNVs in oncogenes and TSGs compared to 100 randomly sampled equally sized sets from common and benign variants preserving the degree distribution of the corresponding cancer sets.

**Supplementary Table S6.** Comparison to annotations with other tools. Methods used and categories assigned are according to Annovar [54] documentation at<http://annovar.openbioinformatics.org/en/latest/user-guide/filter/>. Methods are named and predictions are denoted with one-letter abbreviations according to the Annovar documentation. The first five tabs report comparison of our structural annotation with predictions by other methods within individual biological datasets, the last tab reports comparison between sets of cancer somatic and common nsSNVs. Significant p-values in Fisher's exact test with the significance level < 0.05 are shown in red.

**Supplementary Table S7.** Top 20 ReactomeDB pathways and top 20 GO terms in the “Process” category identified in differential analysis of disease-associated datasets compared to the set of random mutations in random human genes.
