# Supplementary material for: Spatial distribution of disease-associated variants in three-dimensional structures of protein complexes
Source: Oncogenesis. 2017 Sep 25;6(9):e380–. doi: 10.1038/oncsis.2017.79 (PMC5623905; doi:10.1038/oncsis.2017.79)
Supplement: Supplementary Fiugres [file oncsis201779x2.pdf]

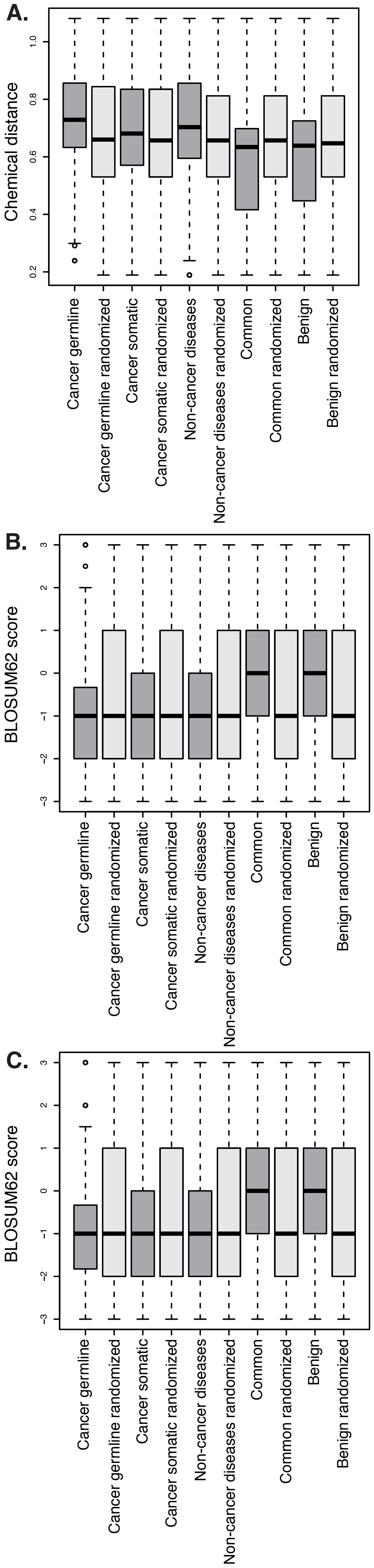


Supplementary Figure S1.

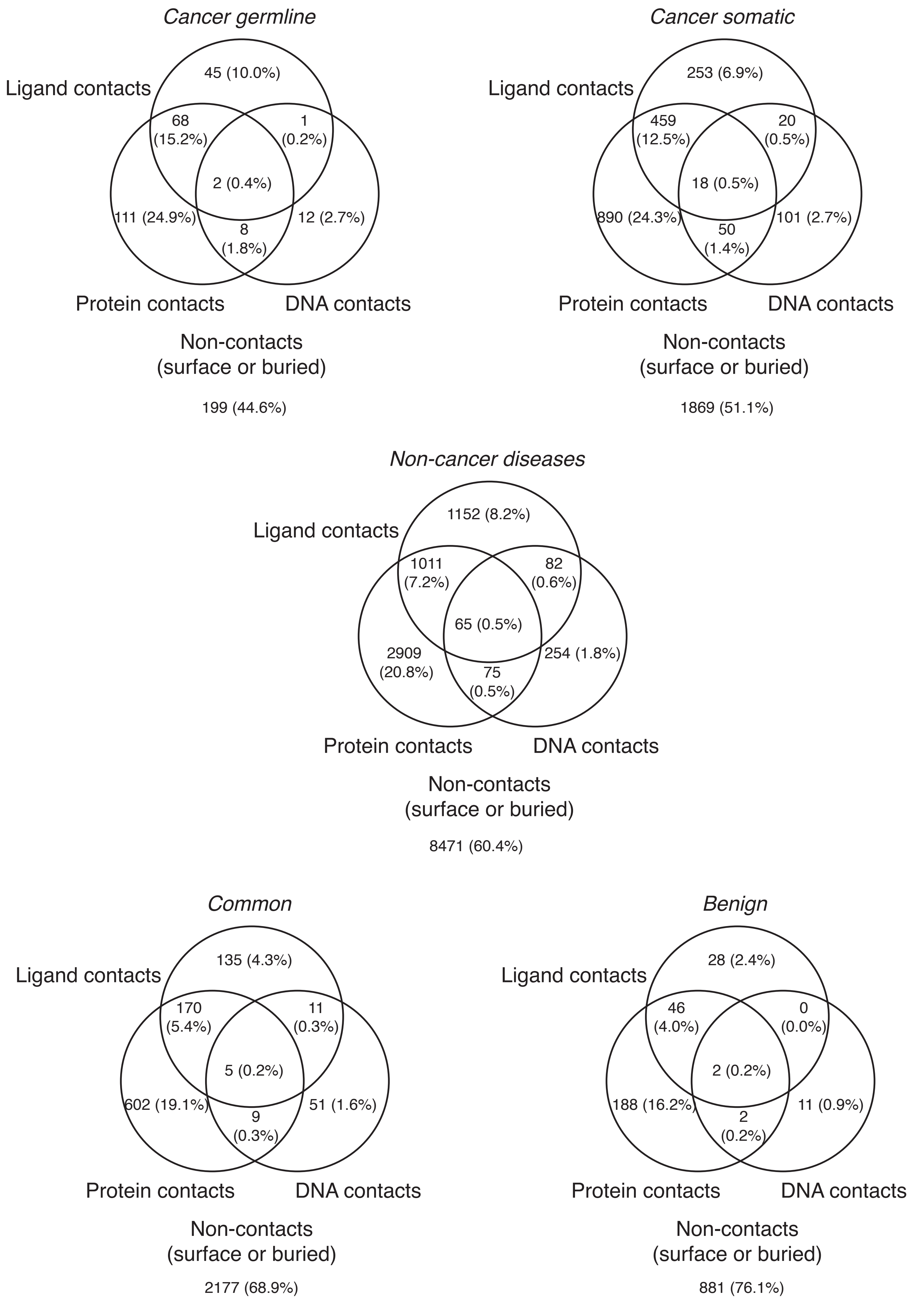


Supplementary Figure S2.

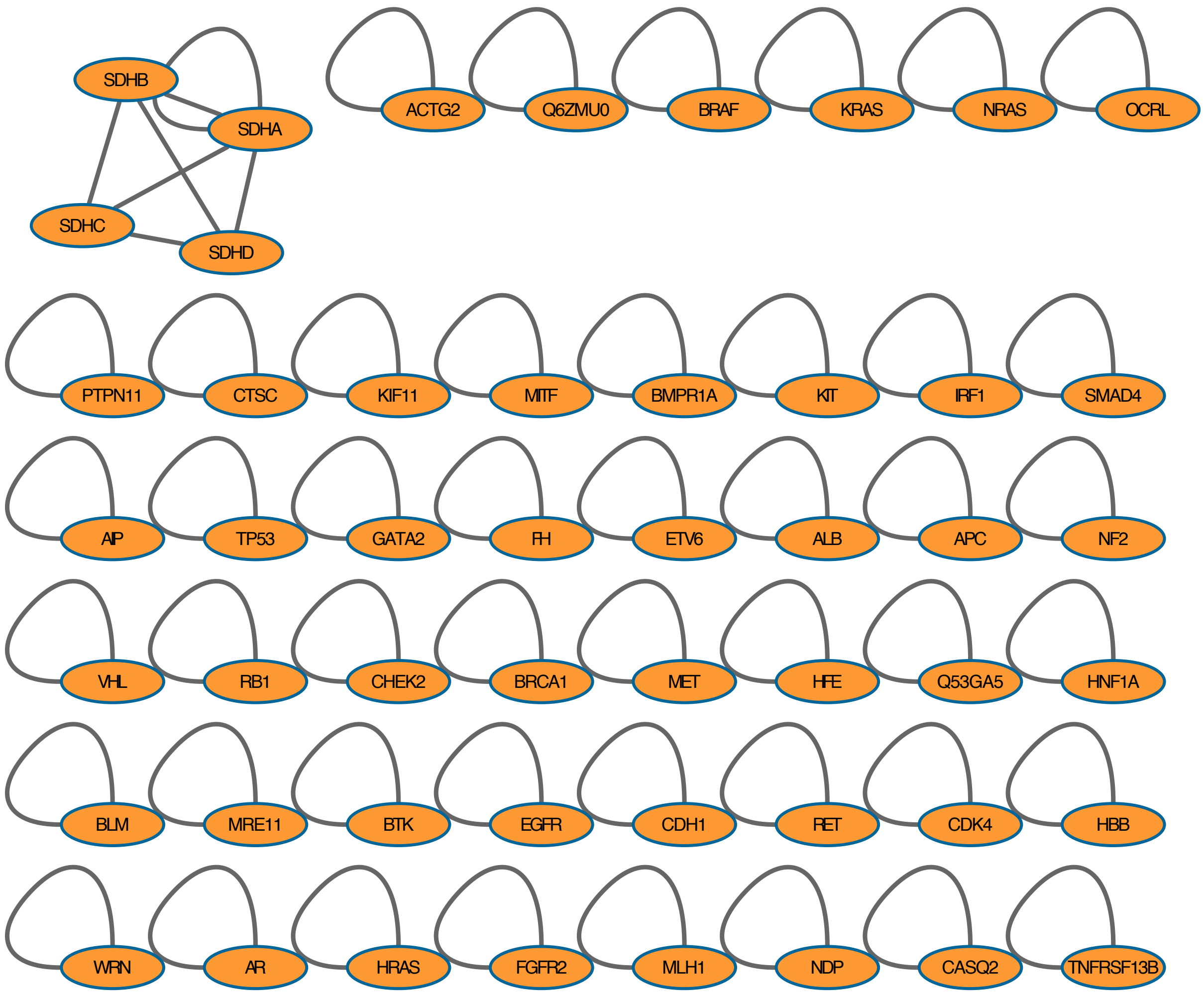


Supplementary Figure S3.

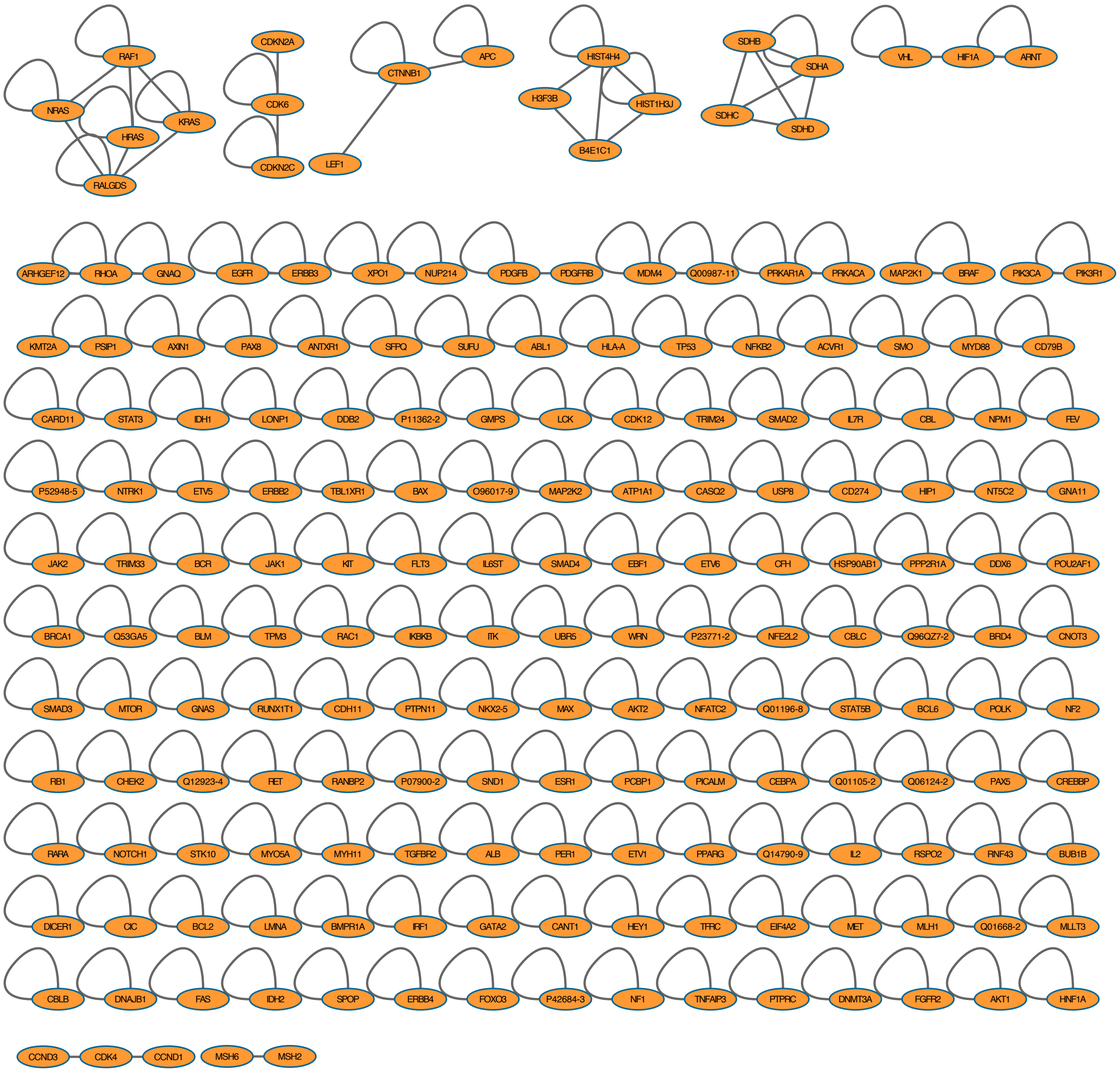


Supplementary Figure S4.

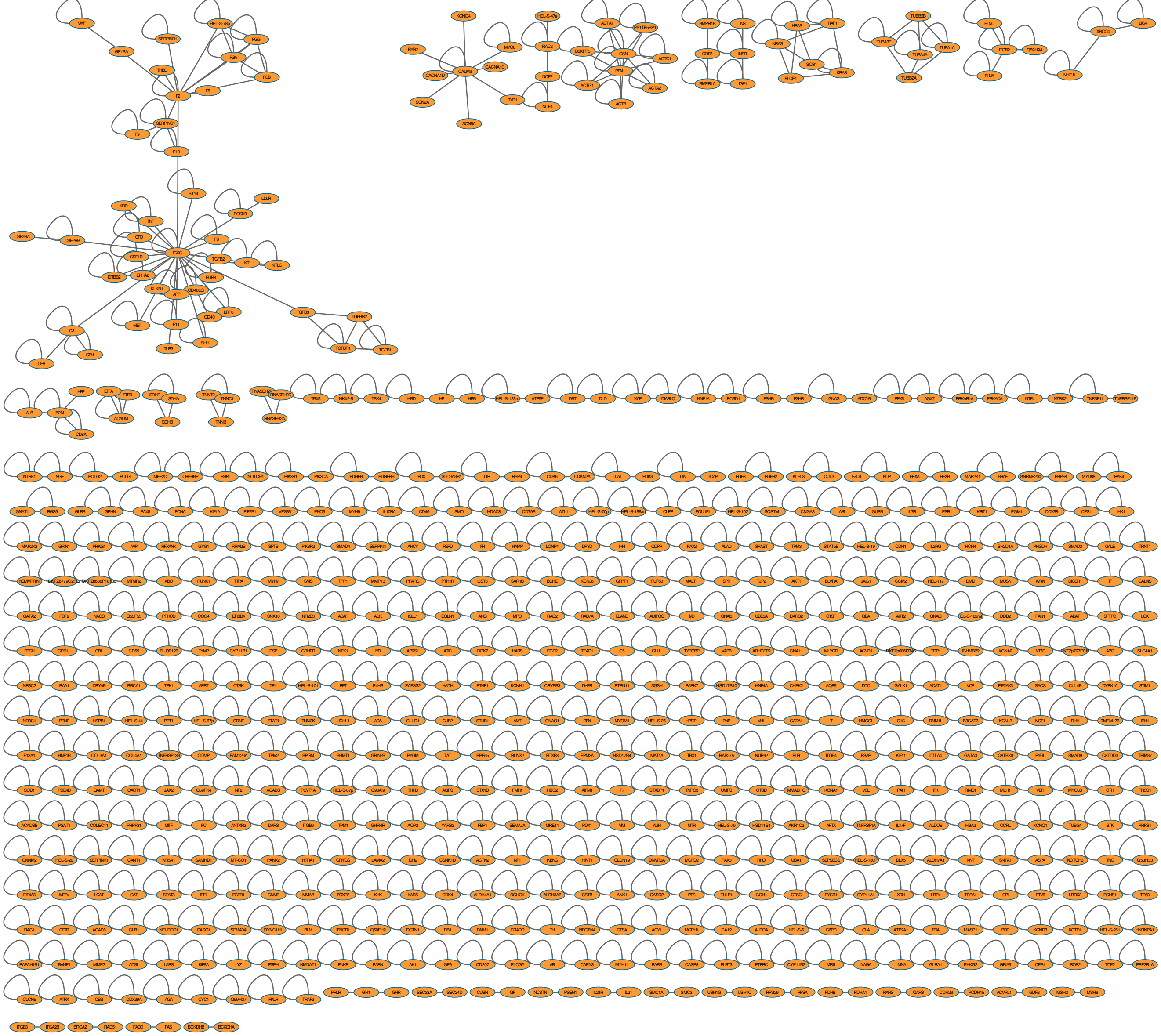

Supplementary Figure S5.

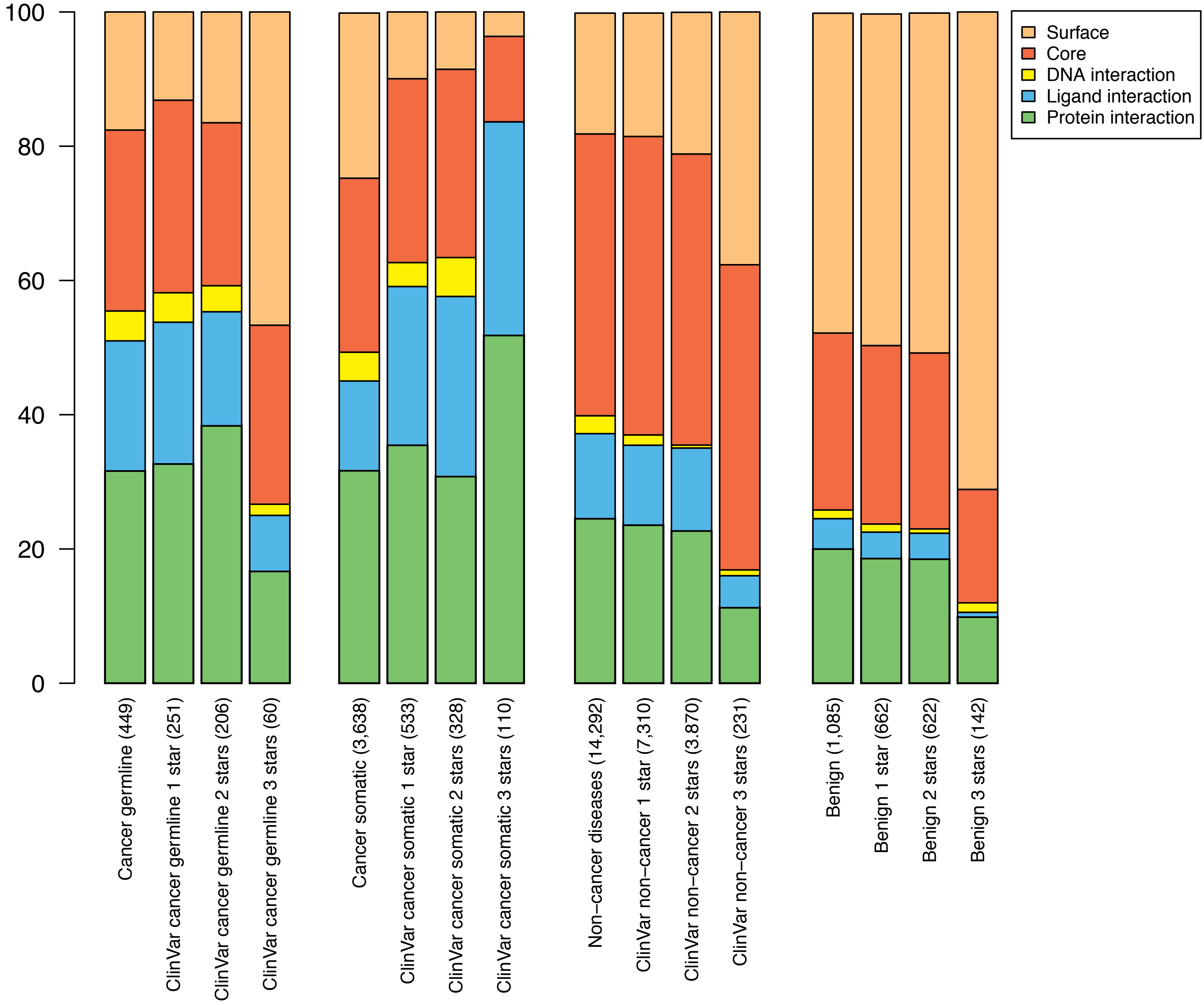


Supplementary Figure S6.
